# Combining the strengths of radiologists and AI for breast cancer screening: a retrospective analysis

**DOI:** 10.1016/S2589-7500(22)00070-X

**Published:** 2022-07

**Authors:** Christian Leibig, Moritz Brehmer, Stefan Bunk, Danalyn Byng, Katja Pinker, Lale Umutlu

**Affiliations:** Vara, Berlin, Germany; Vara, Berlin, Germany; Department of Diagnostic and Interventional Radiology and Neuroradiology, University-Hospital Essen, Essen, Germany; Vara, Berlin, Germany; Vara, Berlin, Germany; Department of Radiology, Breast Imaging Service, Memorial Sloan Kettering Cancer Center, New York, NY, USA; Department of Biomedical Imaging and Image-Guided Therapy Division of Molecular and Gender Imaging, Medical University of Vienna, Vienna, Austria; Department of Diagnostic and Interventional Radiology and Neuroradiology, University-Hospital Essen, Essen, Germany

## Abstract

**Background:**

We propose a decision-referral approach for integrating artificial intelligence (AI) into the breast-cancer screening pathway, whereby the algorithm makes predictions on the basis of its quantification of uncertainty. Algorithmic assessments with high certainty are done automatically, whereas assessments with lower certainty are referred to the radiologist. This two-part AI system can triage normal mammography exams and provide post-hoc cancer detection to maintain a high degree of sensitivity. This study aimed to evaluate the performance of this AI system on sensitivity and specificity when used either as a standalone system or within a decision-referral approach, compared with the original radiologist decision.

**Methods:**

We used a retrospective dataset consisting of 1 193 197 full-field, digital mammography studies carried out between Jan 1, 2007, and Dec 31, 2020, from eight screening sites participating in the German national breast-cancer screening programme. We derived an internal-test dataset from six screening sites (1670 screen-detected cancers and 19 997 normal mammography exams), and an external-test dataset of breast cancer screening exams (2793 screen-detected cancers and 80 058 normal exams) from two additional screening sites to evaluate the performance of an AI algorithm on sensitivity and specificity when used either as a standalone system or within a decision-referral approach, compared with the original individual radiologist decision at the point-of-screen reading ahead of the consensus conference. Different configurations of the AI algorithm were evaluated. To account for the enrichment of the datasets caused by oversampling cancer cases, weights were applied to reflect the actual distribution of study types in the screening programme. Triaging performance was evaluated as the rate of exams correctly identified as normal. Sensitivity across clinically relevant subgroups, screening sites, and device manufacturers was compared between standalone AI, the radiologist, and decision referral. We present receiver operating characteristic (ROC) curves and area under the ROC (AUROC) to evaluate AI-system performance over its entire operating range. Comparison with radiologists and subgroup analysis was based on sensitivity and specificity at clinically relevant configurations.

**Findings:**

The exemplary configuration of the AI system in standalone mode achieved a sensitivity of 84·2% (95% CI 82·4–85·8) and a specificity of 89·5% (89·0–89·9) on internal-test data, and a sensitivity of 84·6% (83·3–85·9) and a specificity of 91·3% (91·1–91·5) on external-test data, but was less accurate than the average unaided radiologist. By contrast, the simulated decision-referral approach significantly improved upon radiologist sensitivity by 2·6 percentage points and specificity by 1·0 percentage points, corresponding to a triaging performance at 63·0% on the external dataset; the AUROC was 0·982 (95% CI 0·978–0·986) on the subset of studies assessed by AI, surpassing radiologist performance. The decision-referral approach also yielded significant increases in sensitivity for a number of clinically relevant subgroups, including subgroups of small lesion sizes and invasive carcinomas. Sensitivity of the decision-referral approach was consistent across the eight included screening sites and three device manufacturers.

**Interpretation:**

The decision-referral approach leverages the strengths of both the radiologist and AI, demonstrating improvements in sensitivity and specificity surpassing that of the individual radiologist and of the standalone AI system. This approach has the potential to improve the screening accuracy of radiologists, is adaptive to the requirements of screening, and could allow for the reduction of workload ahead of the consensus conference, without discarding the generalised knowledge of radiologists.

**Funding:**

Vara.

## Introduction

The rise in popularity of deep neural networks (DNNs) in medical imaging, triggered by advances in artificial intelligence (AI) for image recognition and the increased availability of digital mammography data, have elicited interest in new models based on quantitative imaging features for improved mammography interpretation.^1^ Newly-published studies on DNN-based detection and classification of lesions on the basis of digital mammography data have shown that such systems have comparable diagnostic performance to radiologists and are promising as decision support systems,^2–8^ but the current evidence is insufficient to judge accuracy within breast-cancer screening programmes.^9^

Previous work has demonstrated the potential of combining the strengths of radiologists and machine learning models using ensemble learning methods, consolidating predictions from radiologists and models.^7,8^ However, a major drawback of such an approach is the necessity for the radiologist to evaluate all studies, and thus the workload of the radiologist is not alleviated by AI. Other work has evaluated an AI-powered triaging approach for screening, whereby exams with a high probability of being cancer free are triaged and the remaining exams are referred to the radiologist.^10–15^ These studies, however, showed that sizable reductions of screening exams from the radiologist workload might come at an unacceptable reduction of sensitivity. One commercial solution incorporated normal triaging in one step followed by the identification of women at risk of false negatives who had a negative double reading, but who could benefit from enhanced assessment with supplemental imaging with MRI or ultrasound.^10^ Although this approach indirectly improved the sensitivity of cancer screening, it only focused on predictions on the future risk of interval cancer or next-round screen-detected cancers missed by both readers, and it did not focus on predictions on cancer-positive exams visible on mammography at screening itself, which might be missed by one of two readers in a double-reader setting. Therefore, to date, no study has explored AI combining normal triaging and cancer detection at the point of mammography screen reading by individual radiologists, ahead of the consensus conference, and the effect of such an approach on the sensitivity and specificity of the radiologist. Understanding how such an AI system could affect radiologist screening metrics requires an illustration of how these two systems must work together to achieve joint improvement of sensitivity and specificity, given that increased sensitivity usually comes at the expense of reduced specificity, and vice versa.

In search of an AI-based system that can be used by individual readers ahead of consensus or arbitration meetings, simultaneously improves reader sensitivity, and maintains or even improves specificity while safely triaging normal studies, we propose an AI system that uses a decision-referral approach. This decision-referral approach performs very confident algorithmic assessments automatically, whereas less confident assessments are referred to the radiologist. This two-part system incorporates triage of normal exams while also introducing a safety net to maintain a high degree of sensitivity by performing predictions on cancer-positive exams. This system is intended to be used by the individual radiologist reading the screening mammogram, before consensus review, and therefore, evaluation of its performance on screen-detected cancers and follow-up-proven normal mammography exams is warranted.

To improve diagnostic performance, we first have to show that the confident^16^ predictions of the model, which would enable these studies to be assessed fully automatically without being referred to the radiologist, would outperform those of the human reader. Here, we describe the development and evaluation of such a DNN-based cancer-classification algorithm, using a dataset of 1 193 197 screening studies derived from a national breast-cancer screening programme. We hypothesised that the model would be sensitive and specific enough to independently triage normal cases and recognise suspicious cases. Moreover, we aimed to demonstrate improvement of screening diagnostic accuracy (sensitivity and specificity) of the radiologist when using the decision-referral approach, with generalisability across different screening sites and device manufacturers. The performance of the decision-referral approach was further contrasted with the performance of the AI algorithm in standalone mode.

## Methods

### Study design

**Figure 1:**
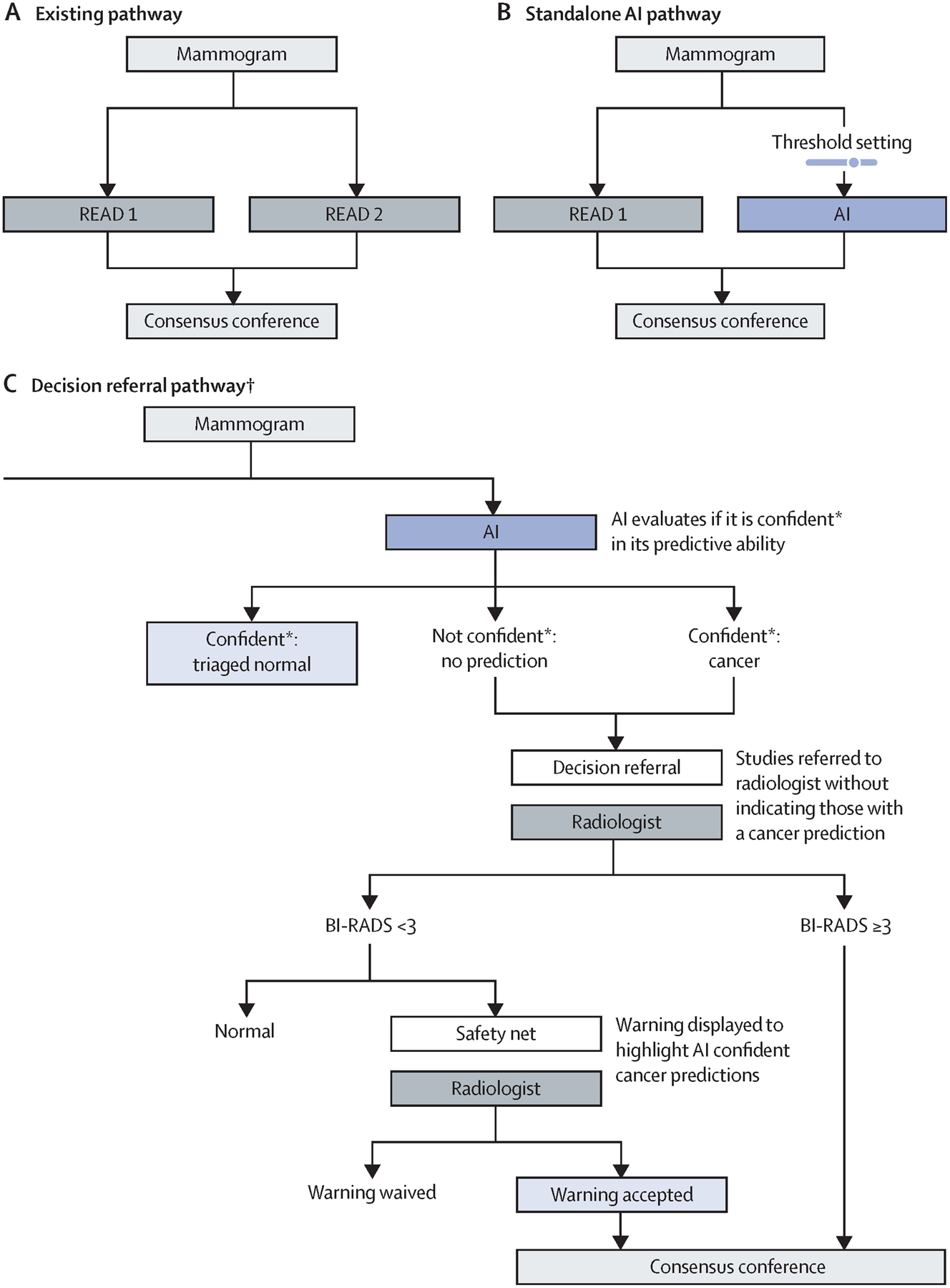
Comparison between the decision-referral and standalone AI pathway in double-reader screening settings Different possible screening pathways are presented. (A) The existing screening pathway, in which mammography studies are independently reviewed by two readers and discordant findings are resolved during consensus. (B) The standalone AI pathway, the most commonly proposed implementation pathway for AI systems. Standalone is defined by the taking over of all decisions from one radiologist, sometimes also referred to as an independent read. (C) The decision-referral pathway, which is the focus of this evaluation. All mammography studies are first read by the AI system, and predictions are produced. AI=artificial intelligence. *The model exhibits a score between 0·0 and 1·0 indicating the malignancy of a study. Scores lower than the threshold for negative predictions (triaged as normal) or higher than the threshold for positive predictions (safety net) were considered confident. All other scores between the two thresholds were not considered confident and the corresponding studies were referred to the radiologist. †Decision-referral approach when used by a single reader in a double reader setting.

In this retrospective analysis study, the screening performance of a single unaided radiologist based on their original clinical decisions in the screening programme (figure 1A) was compared with that of a standalone AI system (figure 1B) and a decision-referral approach (figure 1C) that pairs normal triage and cancer detection via a safety-net warning system. The decision of the original radiologist were those recorded during clinical practice without AI support at the point of screen reading before consensus conference or arbitration. Therefore, the analyses in this study were restricted to screen-detected cancers and follow-up-proven normal mammography exams.

We simulated a screening scenario (figure 1C) in which, in a first step, the AI system classified whether a study was normal or suspicious for cancer and provided at the same time an indication of its confidence on its classification, on the basis of two thresholds.^17^ Both suspicious studies and studies for which the algorithm was unconfident and required human interpretation were referred to the radiologist without indication of the AI-system classifications. We further evaluated a safety net, which was triggered by studies deemed confidently suspicious for cancer by the AI system.

### Simulation assumptions

Because of the retrospective nature of this study, we evaluated the sensitivity and specificity for cancer detection drawn from the scenario in which the radiologist accepts the AI model’s classifications of triaged normal and safety-net studies, whereas the classifications of the remaining studies were based on the radiologist’s decisions. This is equivalent to modelling the confident AI predictions as fully automatic (ie, no AI predictions need to be shown to the radiologist), and thus allowed us to avoid hypothesising about and accounting for human–AI interactions.

### Data sources

This study was reviewed by data privacy lawyers to ensure compliance with the EU General Data Protection Regulation. Ethical approval and the need to obtain informed consent were waived for this study under regional and national law because of the retrospective and fully anonymised nature of the mammography studies and patient data.

**Figure 2:**
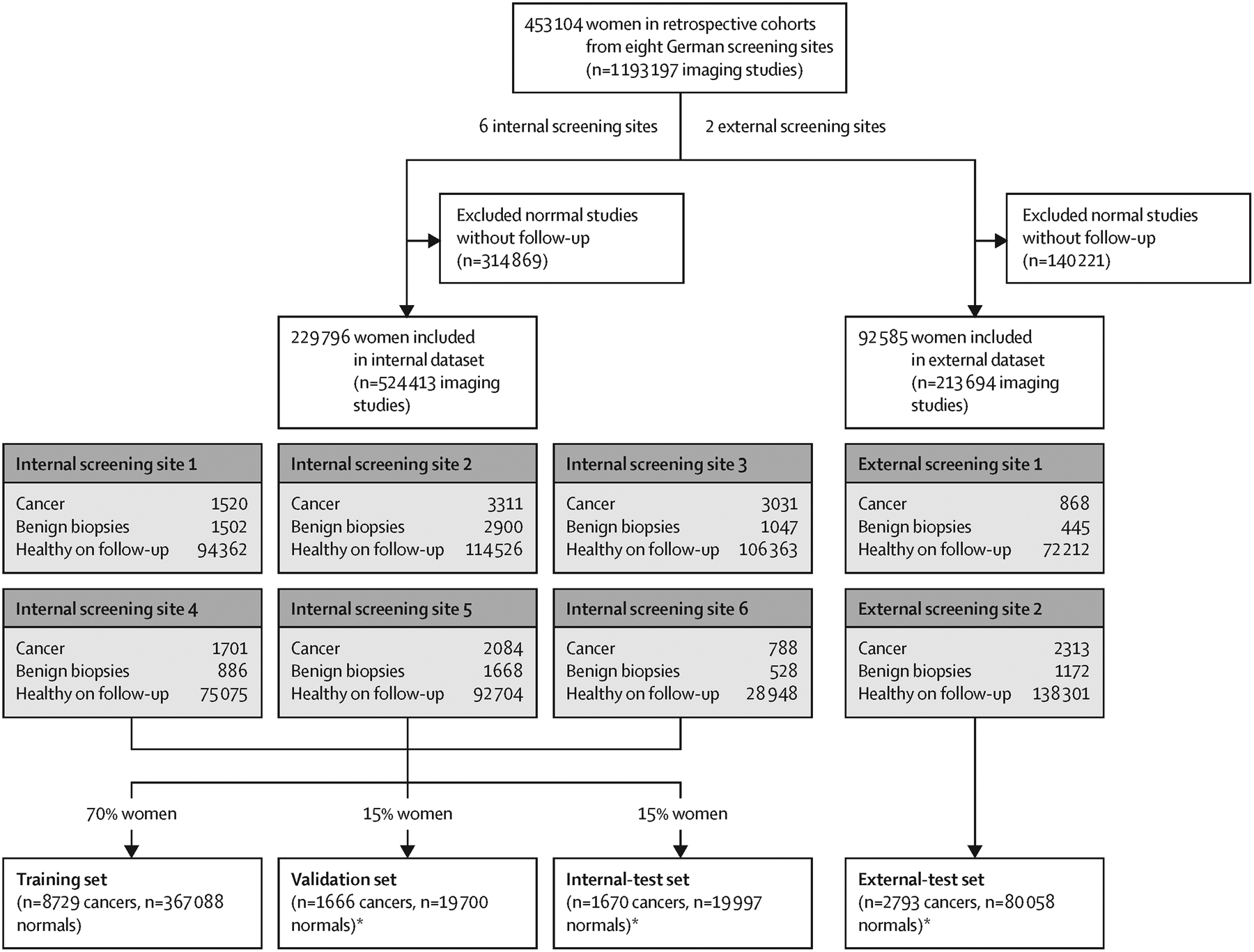
Dataset partitions Further information about study inclusion criteria, the German national breast-cancer screening programme, and the sample weighting technique is available in the appendix (p 6). *Subsample normal mammography exams, one study per woman.

We used a retrospective dataset consisting of 1 193 197 full field, digital mammography studies carried out between Jan 1, 2007, and Dec 31, 2020, from 453 104 women, data which were retrieved from eight German screening sites. We derived an internal-test dataset from six screening sites, and an external-test dataset of breast cancer screening exams from two additional screening sites (figure 2). All mammography studies were done for screening purposes in women who were asymptomatic presenting to the national breast screening programme; no diagnostic or recall images were used. Suspicious studies that went into the consensus conference, including those which were recalled and biopsied, were oversampled during data collection, but this enrichment was addressed during model evaluation with a weighting approach described in the statistical analysis section. All cancers in the dataset were detected by screening; cancers missed or diagnosed in the interval between screening rounds were not included. Normal mammography exams were derived from women with follow-up screening within a minimum of 24 months, which were not recalled (BI-RADS 1 or 2) or in the case of a finding, the follow-up study must have been deemed negative either by double read, consensus conference, or negative recall (appendix p 2). All studies comprised four standard views, bilateral craniocaudal and mediolateral oblique. Regarding device manufacturers, 43·1% of the mammography studies were obtained using a Siemens device, 36·2% a Hologic device, and 8·4% a Fuji device. The remaining 12·3% of mammography devices were obtained using devices made by other manufacturers; these were included in the training dataset but were excluded from subsequent evaluations. Women were 50–70 years of age at screening; more than 80% of women were assigned breast-density categories American College of Radiology (ACR) B or C (appendix p 2).

Data obtained across six screening sites were used as an internal dataset, randomly split by patient ID into training, validation, and test datasets, following the standard practice for developing and evaluating machine learning models.^18^ Each split was mutually exclusive; therefore, women whose data were used for model training (70%) and validation (15%) were not included in the test dataset (15%). The training and validation datasets were used to develop the AI system. Validation data was used to configure the decision-referral thresholds (appendix p 3).

We used two datasets to evaluate the performance of the algorithm, the internal-test dataset, and the external-test dataset. The internal-test dataset constituted an independent sample of women who were not included in the training or validation datasets, although they were from the same six screening sites used to develop the algorithm. To verify that the achieved performance of the algorithm was not caused by shortcut learning^19^ from signals specific to those six screening sites, but rather generalised to different screening sites, we supplemented this evaluation with an evaluation on out-of-distribution data from two additional screening sites previously unseen by the AI system. To account for the enrichment of each dataset caused by oversampled cancer cases, we used a weighting technique^20,21^ to ensure the test datasets were reflective of a real screening population (appendix p 6).

### Development of the AI algorithm

The AI algorithm classifies cancer on a study level. Only study-level labels and predictions are needed for evaluation of the feasibility of decision referral. Whether the decision-referral approach can improve screening metrics depends on whether the model can make better predictions than radiologists on a subset of studies. We present a model based on a deep convolutional neural network, trained with mammography images using labels across different scales (patch, image, and study) for training purposes only. Those labels were derived from annotations of radiological findings and associated biopsy information.

Imaging findings that were biopsy-confirmed were annotated by board-certified radiologists. These comprised radiological findings that were initially classified as suspicious (BI-RADS 4 or 5, ie, suspicious or highly suspicious of malignancy) and that were later recommended for biopsy on assessment, and radiological findings that were initially classified as BI-RADS 2 or 3 and that later underwent biopsy per the patients’ preference. The radiologists used a dedicated web-based radiology viewer, allowing them simultaneous access to histopathology and radiology reports. Data regarding the histopathological reference standard were extracted from reports stored in the official screening software of the German screening programme. Reports were standardised according to the fourth edition of the European Guidelines for Quality Assurance in Breast Cancer Screening and Diagnosis.^22^ Studies were labelled as positive on the basis of histopathological confirmation.^23,24^ Radiologists segmented each suspicious region in the respective images with a polygon. Model architecture and training is described in the appendix (p 8).

### Evaluation of the AI algorithm

The internal-test dataset contained 1670 biopsy-confirmed screen-detected cancer cases and 19 997 follow-up-proven normal mammography exams, whereas the external-test dataset contained 2793 screen-detected cancers and 80 058 follow-up-proven normal mammography exams. These datasets were used to evaluate the performance of the standalone AI approach (figure 1B) and the decision-referral approach (figure 1C). Standalone AI refers to the AI taking over all decisions from the radiologist (ie, no decisions are referred). The decision-referral approach combines confident algorithmic predictions that are not referred to the radiologist with the referral of less confident studies to the radiologist, with the hypothesis that this strategy maintains or improves upon key screening metrics, as the AI features of a safety net and normal triaging achieves a complementary overall improvement of the sensitivity and specificity of the radiologist. The decision-referral approach naturally transitions into standalone mode when all algorithmic predictions are considered confident. Radiologist sensitivity refers to the number of screen-detected cancers found by the individual radiologist, divided by the total number of screen-detected cancers in the dataset, which is the sensitivity as a percentage of double-reading sensitivity, with two readers finding 100% of screen-detected cancers.

### Configuration of the decision referral and standalone AI approaches

The decision-referral approach was configured as follows: lower thresholds (appendix p 4) for confident negative (normal triaging) and upper thresholds for positive (safety-net) predictions were set such that the desired sensitivity and specificity was achieved on the validation dataset (figure 2). Given two thresholds, we computed the overall sensitivity and specificity of the combined system on the basis of the AI assessments on confident studies and radiologist assessments on unconfident studies. The resulting sensitivities and specificities on validation data were used to choose the desired sensitivity and specificity trade-off. A clinically meaningful configuration maximizes radiologist sensitivity without decreasing specificity. On the validation dataset, an algorithmic sensitivity of 97% and specificity of 98% was the best trade-off achieved (appendix p 3). The configuration that achieved this sensitivity and specificity is used exemplarily to present the main results here, whereas further configurations are shown in the table. To quantify workload reduction, triaging performance was computed as the rate of studies correctly tagged as normal (ie, the fraction that could be automated).

Standalone AI was configured by setting a single threshold (appendix p 3) such that the radiologist sensitivity of 86% was achieved on the validation dataset (figure 2).

### Statistical analysis

Curves for receiver operating characteristic (ROC) and areas under the ROC (AUROC) were used as metrics to evaluate standalone AI performance over its entire operating range. For given operating points of standalone AI, the radiologist, and the decision-referral approach, estimates of sensitivity and specificity were calculated. For point estimates involving a radiologist decision, the two independent decisions per study were averaged. For error estimates and hypothesis tests, resampling methods were used. For all estimated metrics, 95% CIs were determined on the basis of 1000 bootstrap samples.^25^ Variability of human judgement influences the radiologist and the decision-referral metrics and was accounted for by a two-step sampling procedure as follows: for each mammographic study, one radiologist assessment was sampled from two independent and anonymised readers; and the whole dataset was resampled with replacement.

To understand whether the addition of AI had a consistent effect on sensitivity across clinically relevant subgroups, we calculated subgroup-specific sensitivity values on the internal-test and external-test datasets by different levels of biopsy score, ACR breast density, lesion size, and radiological findings according to BI-RADS.^23,24,26^ Generalisability was similarly assessed by comparing results on the internal validation and test datasets, and by presenting sensitivity stratified across screening sites and device manufacturers, and specificity stratified across device manufacturers.

Differences in sensitivity and specificity of standalone AI versus radiologist and decision referral versus radiologist were assessed for statistical significance using a permutation test.^25^ For each of 10 000 trials, as for the CIs, one of two radiologist decisions were sampled independently for each mammographic study, and each decision-referral decision was randomly permuted with the radiologist decision. A two-sided p value was computed by comparing the observed difference with the quantiles of the null distribution.^3^

Sample weights reflecting the actual distribution of study types in the German breast-screening population applied to validation and test datasets (figure 2) are described in the appendix (p 6).^20,21^ Analyses were done using the Python version 3.8.10 scientific computing stack.

### Role of the funding source

The funder of the study was involved in the collection, management, and analysis of the data used to develop the AI algorithm, and in the preparation and review of the manuscript. The authors not employed by the funder had control of the data and information submitted for publication at all times and made the final decision to submit the manuscript for publication.

## Results

**Figure 3:**
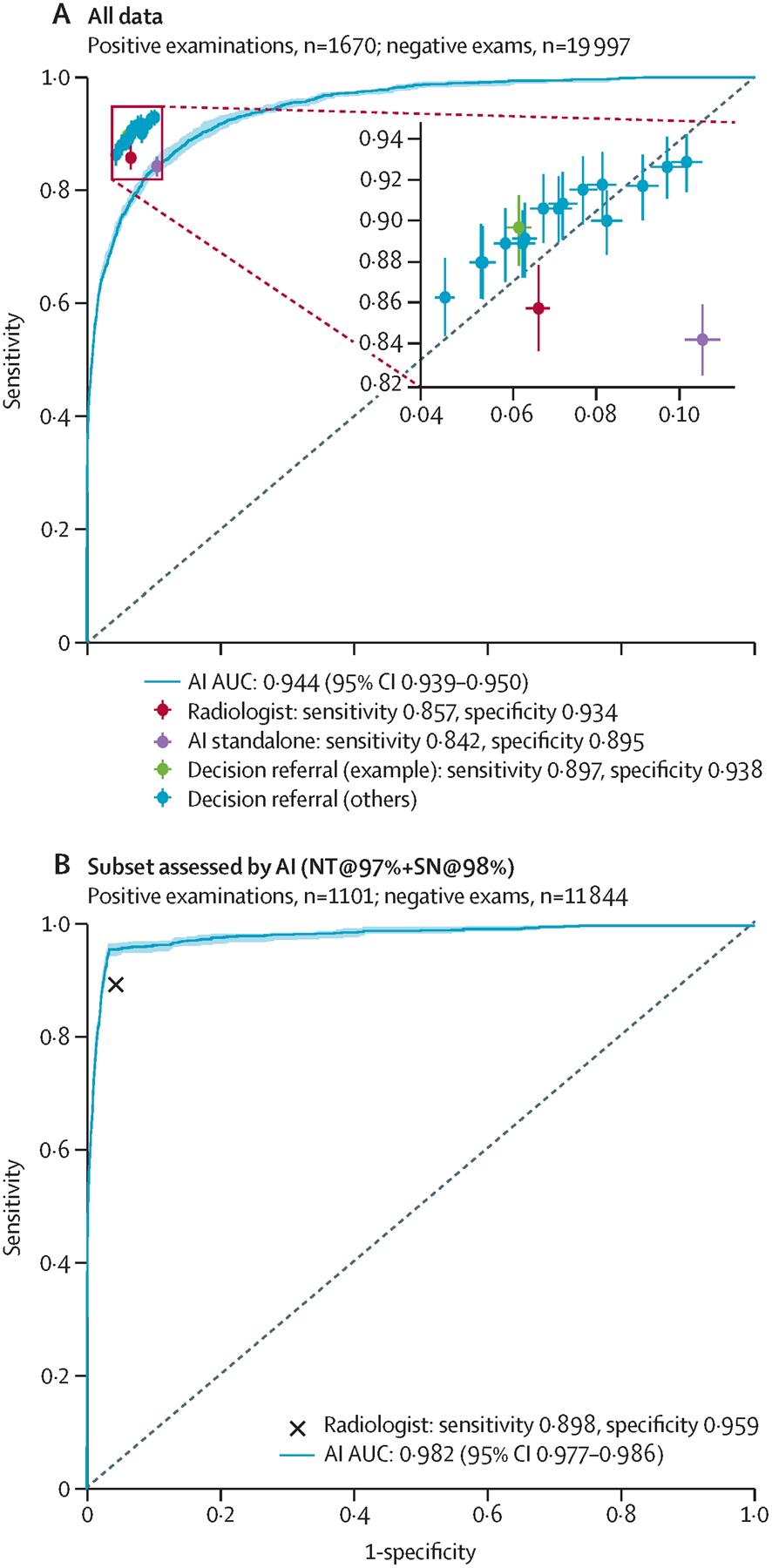
Comparison of the performance of standalone and decision-referral approaches based on the internal-test dataset Overall screening diagnostic accuracy for radiologists, standalone AI, and decision referral are presented. Sensitivity and specificity are given for radiologists (red), standalone AI (purple), and decision referral (green for the exemplary configuration NT@97%+SN@98% and blue for alternative configurations). In addition, we present ROC curves and AUROC to evaluate AI-system performance over its entire operating range on the internal-test dataset (n=21 667; A) and on the subset of data for which it is able to produce its most confident predictions for the exemplary configuration NT@97%+SN@98% (B). Error bars denote 95% CIs. The decision-referral approach outperformed the independent radiologist on either or both sensitivity and specificity depending on the configuration (A) by surpassing the radiologist throughout on the confident set of predictions (B). The resulting sensitivity and specificity values for all studies were similar or greater than the radiologist alone, whereas 42·1–71·1% of studies were able to be safely triaged. AI=artificial intelligence. AUC=area under the curve. AUROC=area under the receiver-operating characteristic. NT=normal triage. ROC=receiver-operating characteristic. SN=safety net.

**Figure 4:**
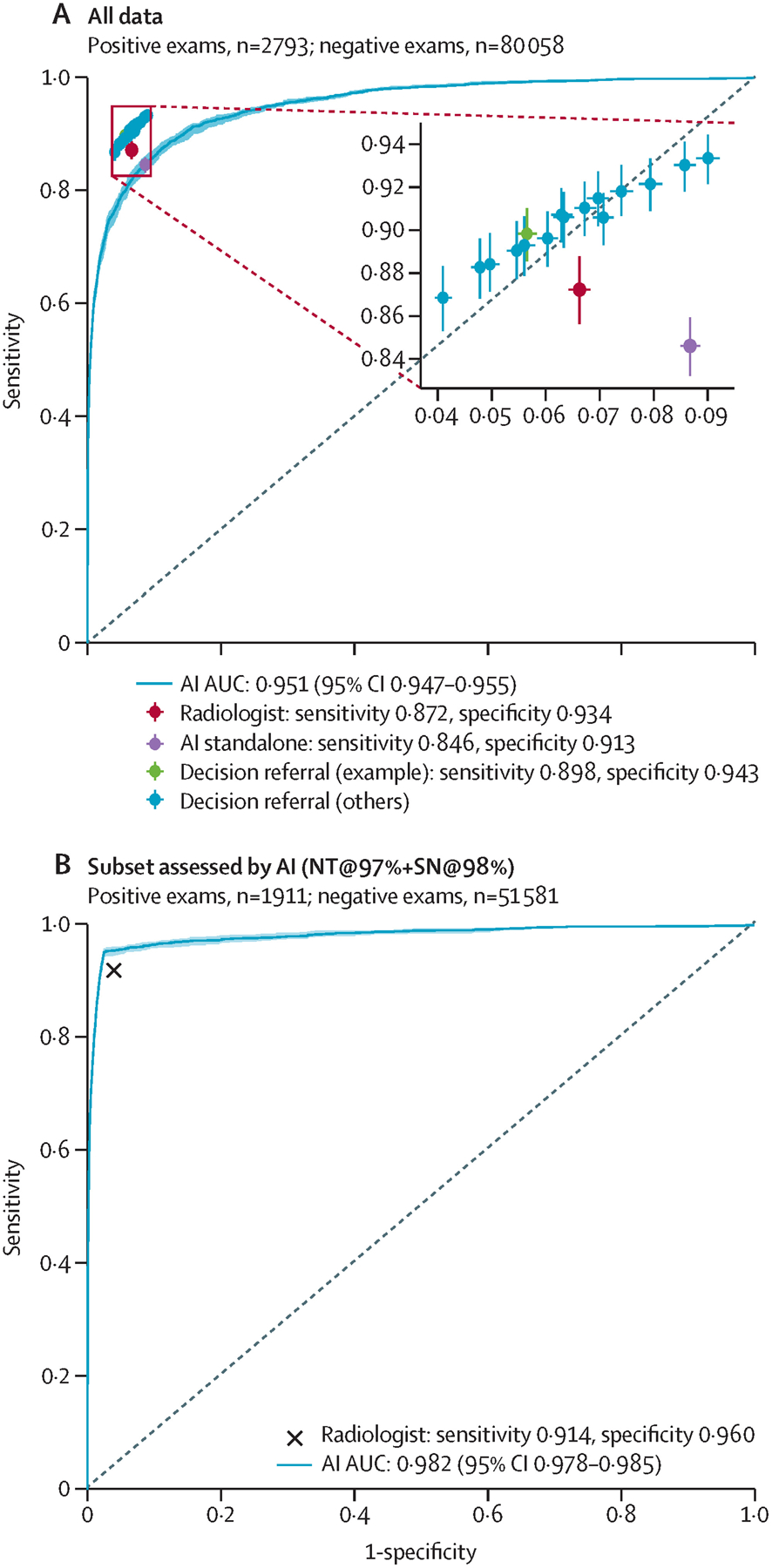
Comparison of the performance of standalone and decision-referral approaches based on the external-test dataset Overall screening diagnostic accuracy for radiologists, standalone AI, and decision referral are presented. Sensitivity and specificity are given for radiologists (red), standalone AI (purple), and decision referral (green for the exemplary configuration NT@97%+SN@98% and blue for alternative configurations). In addition, we present ROC curves and AUROC to evaluate AI-system performance over its entire operating range on the external-test set (n=82 851; A) and on the subset of data for which it is able to produce its most confident predictions for the exemplary configuration NT@97%+SN@98% (B). Error bars denote 95% CIs. The decision-referral approach outperformed the independent radiologist on either or both sensitivity and specificity depending on the configuration (A) by surpassing the radiologist throughout on the confident set of predictions (B). The resulting sensitivity and specificity values for all studies were similar or greater than the radiologist alone, whereas 44·5–73·8% of studies were able to be safely triaged. AI=artificial intelligence. AUC=area under the curve. AUROC=area under the receiver-operating characteristic. NT=normal triage. ROC=receiver-operating characteristic. SN=safety net.

The performance of the standalone AI system is contrasted with the performance of the radiologist (figure 3A, 4A). The performance of the standalone AI system across all possible configurations is shown by the corresponding ROC curve, reaching an AUROC of 0·944 (95% CI 0·939–0·950) on the the internal-test dataset, and 0·951 (0·947–0·955) on the the external-test dataset. On the internal-test dataset, the radiologist achieved a sensitivity of 85·7% (95% CI 83·6–87·9) and a specificity of 93·4% (95% CI 93·1–93·7), compared with a sensitivity of 84·2% (82·4–85·8) and a specificity of 89·5% (89·0–89·9) for the operating point of the standalone AI system that maintained radiologist sensitivity on the validation dataset (figure 3A, 4A; table). On the external-test dataset, radiologist performance compared with standalone AI was 87·2% (85·6–88·7) versus 84·6% (83·3–85·9) on sensitivity, and 93·4% (93·2–93·6) versus 91·3% (91·1–91·5) on specificity. The sensitivity and specificity of the standalone AI system was significantly lower than the unaided radiologist on both test datasets (p=0·0019 for external-test data sensitivity and p<0·0001 for internal-test and external-test data specificity), but sensitivity was not significantly different on the internal-test dataset (p=0·17).

The performance of the decision-referral approach is plotted with crosshairs (figures 3A, 4A). Using the exemplary configuration, the decision-referral approach achieved a sensitivity of 89·7% (87·9–91·3) and specificity of 93·8% (93·6–94·1), which represented a 4·0 percentage point improvement on sensitivity and 0·5 percentage point improvement on specificity compared with the unaided radiologist on the internal-test dataset (table). This finding corresponded to a triaging performance at 60·7%, and a statistically significant improvement of both sensitivity and specificity (sensitivity p<0·0001; specificity p=0·0002). On the external-test dataset, the decision-referral approach similarly achieved a significant improvement on both sensitivity (2·6 percentage points) and specificity (1·0 percentage point; p<0·0001 for both), corresponding to a triaging performance at 63·0%.

Other possible configurations are shown (figures 3A, 4A; table). The decision-referral approach outperformed the unaided radiologist on both sensitivity and specificity. Configurations for which the decision referral had a different effect on sensitivity and specificity are also shown. Resulting values were similar or greater than for the unaided radiologist, and 42·1–73·8% of studies could be safely triaged.

The AI system performance on the subset of data for which it produced its most confident predictions is shown (figures 3B, 4B). With an AUROC of 0·982 (95% CI 0·977–0·986) on the internal-test dataset and 0·982 (0·978–0·985) on the external-test dataset, the performance of the AI system surpassed the performance of the radiologist.

**Figure 5:**
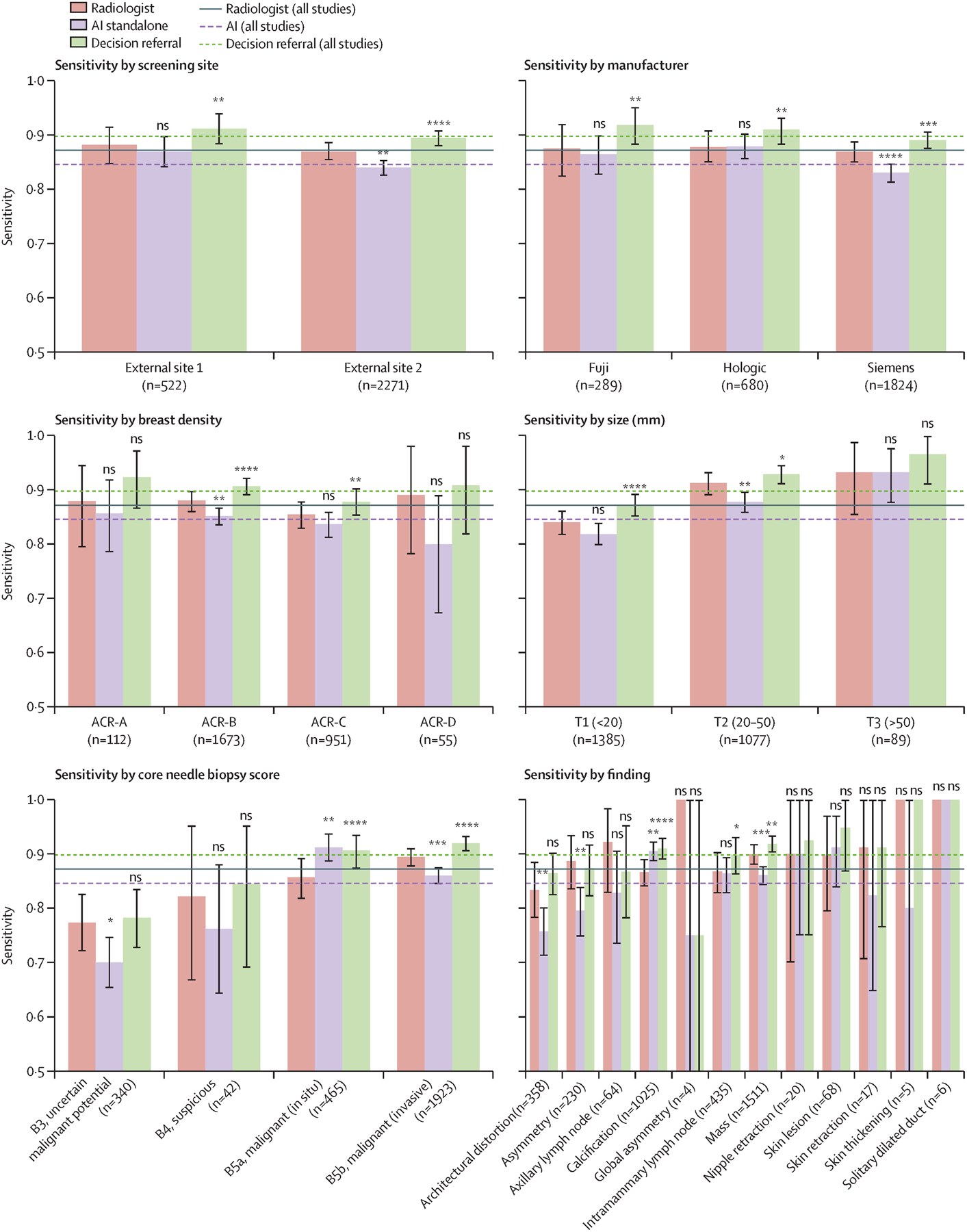
Subgroup performance on sensitivity at exemplary configuration on external-test data Average sensitivities for exemplary configurations of the decision-referral approach (dashed green line, NT@0·97+SN@0·98), are higher than both the average radiologist sensitivity (solid red line) and standalone AI average sensitivity (dashed purple line, configuration as in table). Bar plots show sensitivities stratified across relevant subgroups. Accompanying values are available in the appendix (p 9). AI=artificial intelligence. ns=not significant. NT=normal triaging. SN=safety net. ****p≤0·001. ***p≤0·001. **p≤0·01. *p≤0·05.

The respective average sensitivities of the standalone AI system and of the decision-referral approach across different subgroups are shown by dotted and dashed horizontal lines (figure 5, for the external-test dataset; appendix p 12 for the internal-test dataset). Performance differed across different clinical subgroups. When the average specificity was kept constant, the reduced average sensitivity for standalone AI resulted in negative changes in sensitivity for several clinically relevant subgroups. By contrast, the introduction of decision referral resulted in significant positive changes in sensitivity for several clinically relevant subgroups. The introduction of decision referral improved the ability of the radiologist to detect malignant in-situ and invasive lesions (external-test dataset +4·9% and +2·5%, p≤0·0001 for both; internal-test dataset +3·8% and +4·1%, p=0·01 and p<0·0001). In subgroups stratified by breast density, decision referral yielded significantly higher sensitivity for breasts classified as ACR B (scattered areas of fibroglandular densities) and C (heterogeneously dense), which represent approximately 80% of all women screened.^27^ Percentage points improved from 1·8% to 4·5% on the external-test dataset and 1·0% to 8.3% on the internal-test dataset. In subgroups stratified by imaging findings, decision referral improved sensitivity across several different subgroups, including for masses and calcifications (external-test dataset +1·9% and +4·4%, p=0·0013 and p<0·0001; internal-test dataset +3·7% and +5·1%, p<0·0001 for both). Decision referral improved sensitivity in all subgroups stratified by lesion size. Unlike for standalone AI, no subgroup exhibited a significant decrease in sensitivity when the decision-referral approach was used. Exact values are provided in the appendix (pp 9, 12, 13).

Across all possible configurations, the algorithm alone generalised from an AUROC of 0·943 (95% CI 0·937–0·949) on the validation dataset (appendix p 3) to an AUROC of 0·944 (0·939–0·950) on the internal-test dataset. Specific configurations differed in terms of their specific generalisability. The standalone AI operating point was chosen to match the radiologist sensitivity on the validation dataset (appendix p 3), at the cost of a specificity reduced by 4·5 percentage points (p<0·0001). On the internal-test dataset, the sensitivity and specificity of the standalone configuration trade-off drifted to a reduction in sensitivity by 1·5 percentage points (p=0·17) and specificity by 3·9 percentage points (p<0·0001). The decision-referral approach requires that for a chosen configuration, sensitivity, and specificity improvements are maintained on a different dataset and the algorithmic assessment on the confident subset of studies surpasses the radiologist performance on these. At the exemplary configuration, the decision-referral approach improved sensitivity by 3·6 percentage points and specificity by 0·4 percentage points on the validation dataset (appendix p 3). Reusing the same configuration, sensitivity improved by 4·0 percentage points and specificity by 0·4 percentage points on the internal-test dataset. For the confident subset of studies, the algorithm reached an AUROC of 0·979 (95% CI 0·974–0·984) on the validation dataset (appendix p 5) and an AUROC of 0·982 (0·978–0·986) on the internal-test dataset (figure 3B), surpassing the unaided radiologist performance on each confident subset.

To assess whether the standalone AI system and the decision-referral approach can be generalised to new screening sites previously unseen by the algorithm, the external dataset was derived from two screening sites with different radiologists and women. The standalone AI system maintained an AUROC of 0·951 (0·947–0·955) across all configurations, and its operating point resulted in a reduction of 2·6 sensitivity percentage points and 2·0 specificity percentage points. Decision referral maintained positive changes by surpassing the radiologist on the confident studies (AUROC 0·982, 0·978–0·986; figure 4B), significantly improving sensitivity by 2·6 percentage points and specificity by 1·0 percentage points (p<0·0001 for both).

When stratifying by device manufacturer and screening site, standalone AI was not able to maintain radiologist sensitivity across all subgroups, whereas decision referral achieved sensitivities that were either not significantly lower or instead significantly higher than those of the unaided radiologist (external-test dataset, figure 5 and appendix p 9; internal-test dataset, appendix pp 12, 13). Furthermore, decision referral maintained or improved radiologist specificity across all device manufacturers (appendix pp 16, 17). Taken together, the algorithm and decision-referral approach showed generalisability across the eight different screening sites and mammography devices from three different manufacturers.

## Discussion

Our results, based on an evaluation of an AI system using retrospectively collected mammographic images of 4463 screen-detected cancers and 100 055 follow-up-proven normal studies, demonstrate the potential applicability of AI via a decision-referral approach, a hybrid triaging and cancer detection approach. The simulation of this decision-referral approach showed that combining the strengths of radiologists and AI could result in marked improvements in the sensitivity and specificity of individual radiologists ahead of the consensus conference. Although use of the AI system in standalone mode on the external-test dataset showed a statistically significant reduction of radiologist sensitivity by 2·6 percentage points and specificity by 2·0 percentage points, the very same models could be used to collaborate with the radiologist in decision-referral mode. In fact, the exemplary configuration of the AI system within a decision-referral approach achieved an improvement of radiologist sensitivity by 2·6 percentage points and specificity by 1·0 percentage point, while automatically triaging 63·0% of the studies. This indicates that the safety net was able to detect cancers that were missed by the first reader, and only detected by the second reader. Decision referral could improve overall sensitivity and specificity, because on the subset of data in which the AI system performed predictions, composed of screen-detected cancers and follow-up-proven negatives, an AUROC of 0·982 surpassing the performance of the unaided radiologist was achieved. A series of alternative configurations of the AI system within a decision-referral approach also achieved improved performance.

We confirmed consistent and improved performance of the decision-referral approach across clinically relevant subgroups as well, including those presenting as challenging cases for radiologists. Sensitivity was also consistent across three different device manufacturers and eight different screening sites. Of note, an AI model, if deployed in clinical practice, also has the potential to be further improved by undergoing training on newly incoming data, ensuring that performance on all subgroups does not degrade.

The decision-referral approach would allow screening programmes to iteratively work towards automating more screening decisions within a safe framework, rather than converting to a fully automated AI system without human oversight. The existing literature on the accuracy of AI systems does not lend support to implementation of standalone applications in clinical practice.^9,28^ A published systematic literature review found that 34 (94%) of 35 studies of AI systems were less accurate than a single radiologist, whereas the few small studies showing greater accuracy of a standalone system were at a high risk of bias and had low generalisability to the clinical context.^9^ In standalone mode, our AI achieved a sensitivity of 84·6% and a specificity of 91·3% on external data, also performing less accurately than the average single radiologist. Clear caveats exist, which hamper the adoption of a standalone system. In settings of low cancer prevalence (ie, screening), the variability of positive predictive values among radiologists results in false positives, requiring additional resources for consensus review and diagnostic testing.^29,30^ Fully automated AI does not ameliorate this challenge; ambiguous AI predictions would still result in large numbers of false positives and increased workload. In contrast to standalone AI approaches, the decision-referral approach only makes decisions on a subset of exams with a high degree of accuracy. With further model improvement, this fraction of accurate decisions is expected to increase.

The decision-referral approach is further differentiated from ensemble modelling and standalone triaging approaches because it combines automated triaging of normal cases and decision referral integrating a safety net for positive case prediction; but regarding the safety net, the model intentionally does not provide upfront access to the predictions on the exams referred to the user to avoid potentially misleading bias. In practice, negative model predictions would be presented as prefilled normal reports and positive model predictions as warnings from the safety net, and would only be shown if a radiologist assigned a BI-RADS score lower than 3. A definitive assessment of the overall performance therefore requires final radiologist decisions after algorithmic suggestions, maintaining final human oversight.

We acknowledge the limitations inherent in evaluating the decision-referral approach in a retrospective setting. The retrospective dataset excluded cases that did not have a normal follow-up within 4·5 years after screening. We believe this is a generous period to capture a diverse cohort of women with differing screening uptake practices, for example, including those who might be non-adherent to the biennial screening guidelines. This approach might, however, result in the exclusion of women who were attending their final screening appointment at age 69 years, or who dropped out of screening entirely.

Our analysis required the assumption that confident predictions are done automatically. AI systems for performance and safety-critical tasks should be tested thoroughly before automated decisions are taken. The role of the radiologist remains central to the decision-referral approach we propose. However, this was a simulation not accounting for human–AI interaction, preventing a direct assessment of how AI-generated recommendations influence the decision making of radiologists. Concretely, the simulation made the assumption that the radiologist would not correct any of the algorithmic suggestions, such that prefilled normal reports were assumed to be accepted even if this leads to a missed cancer, and safety-net warnings were accepted even if they were false positives. With human oversight, erroneous but corrected AI predictions can only lead to a further improvement of screening metrics. For correct and accepted predictions, our findings are reflective of the best possible outcomes. For correct but not accepted AI predictions, the algorithm cannot directly be held accountable, but careful education and monitoring of predictions from normal triaging, safety net, and radiologists should be mandatory for AI providers not to repeat the pitfalls of computer-aided detection systems.^31^ With more accurate, confident AI predictions and referred studies (with a cancer prevalence being similar to the overall population), the decision-referral approach is promising. Ultimately, only prospective evaluations of human and AI interaction on a fully representative cohort of women attending screening would be able to provide direct insights into the influence of the decision-referral approach on radiologist decision making.

A further limitation of this study is that it evaluated the performance of a single reader before the consensus conference using the decision-referral approach. An approach to further reduce workload is to have both readers in the double-reader setting use the decision-referral approach. With a triaging performance higher than 50% achieved for each reader, this approach would result in a total workload reduction of more than 100% (out of 200%), outperforming what a standalone AI solution could achieve by replacing one reader (100%). However, understanding the broader effects of applying the same approach to two readers is important future research undertaking that should also include information on interval cancers.

This study has provided evidence to continue on the path towards widespread and safe clinical adoption of AI-based systems for breast screening. The decision-referral approach leverages the strengths of both the radiologist and the AI algorithm, demonstrating that improvements on sensitivity and specificity can be made that surpass that of the individual radiologist and the standalone AI system even if the same underlying algorithm is used. This approach has potential to improve screening accuracy of radiologists, is adaptive to the (heterogeneous) requirements of screening, and could allow for the reduction of workload through triaging normal studies, without discarding the final oversight of the radiologists.

## Supplementary Material

supplemental

## Figures and Tables

**Table:** Diagnostic accuracy and triaging performance for radiologist, standalone AI, and decision referral at selected configurations for internal-test and external-test datasets, with each row representing one resulting operating point over the whole dataset

	Sensitivity (95% CI)	Specificity (95% CI)	Δ sensitivity		Δ specificity		Triaging performance*
			Change	p value	Change	p value	
**Internal·test data**							
Radiologist	85·7% (83·6–87·9)	93·4% (93·1–93·7)	NA	NA	NA	NA	NA
AI standalone	84·2% (82·4–85·8)	89·5% (89·0–89·9)	−1·5%	p=0·17	−3·9%	p<0·0001	89·5%
NT@0·95+SN@0·99	86·3% (84·1–88·0)	95·6% (95·3–95·9)	0·5%	p=0·43	2·2%	p<0·0001	71·1%
NT@0·95+SN@0·98	88·0% (86·1–89·8)	94·7% (94·4–95·0)	2·2%	p=0·0029	1·3%	p<0·0001	71·1%
NT@0·97+SN@0·99	88·0% (86·1–89·7)	94·8% (94·5–95·0)	2·2%	p=0·0001	1·4%	p<0·0001	60·7%
NT@0·98+SN@0·99	88·9% (87·1–90·7)	94·2% (93·9–94·5)	3·2%	p<0·0001	0·8%	p<0·0001	50·5%
NT@0·95+SN@0·97	88·9% (87·1–90·7)	93·8% (93·4–94·1)	3·2%	p<0·0001	0·4%	p=0·0097	71·1%
NT@0·99+SN@0·99	89·1% (87·3–90·9)	93·7% (93·4–94·0)	3·4%	p<0·0001	0·3%	p=0·0002	42·1%
NT@0·97+SN@0·98†	89·7% (87·9–91·3)	93·8% (93·6–94·1)	4·0%	p<0·0001	0·5%	p=0·0002	60·7%
NT@0·95+SN@0·95	90·0% (88·4–91·6)	91·7% (91·4–92·1)	4·3%	p<0·0001	−1·6%	p<0·0001	71·1%
NT@0·98+SN@0·98	90·6% (88·9–92·1)	93·3% (93·0–93·6)	4·9%	p<0·0001	−0·1%	p=0·33	50·5%
NT@0·97+SN@0·97	90·6% (88·8–92·1)	92·9% (92·6–93·2)	4·9%	p<0·0001	−0·5%	p=0·0006	60·7%
NT@0·99+SN@0·98	90·8% (89·1–92·4)	92·8% (92·5–93·1)	5·1%	p<0·0001	−0·6%	p<0·0001	42·1%
NT@0·98+SN@0·97	91·5% (89·9–93·1)	92·3% (92·0–92·7)	5·8%	p<0·0001	−1·1%	p<0·0001	50·5%
NT@0·97+SN@0·95	91·7% (90·2–93·2)	90·9% (90·5–91·3)	6·0%	p<0·0001	−2·5%	p<0·0001	60·7%
NT@0·99+SN@0·97	91·8% (90·2–93·3)	91·9% (91·5–92·2)	6·0%	p<0·0001	−1·5%	p<0·0001	42·1%
NT@0·98+SN@0·95	92·6% (91·2–94·1)	90·3% (89·9–90·7)	6·9%	p<0·0001	−3·1%	p<0·0001	50·5%
NT@0·99+SN@0·95	92·9% (91·3–94·3)	89·8% (89·4–90·2)	7·2%	p<0·0001	−3·5%	p<0·0001	42·1%
**External·test-data**							
Radiologist	87·2% (85·6–88·7)	93·4% (93·2–93·6)	NA	NA	NA	NA	NA
AI standalone	84·6% (83·3–85·9)	91·3% (91·1–91·5)	−2·6%	p=0·0019	−2·0%	p<0·0001	91·3%
NT@0·95+SN@0·99	86·8% (85·3–88·3)	95·9% (95·7–96·1)	−0·4%	p=0·45	2·5%	p<0·0001	73·8%
NT@0·95+SN@0·98	88·3% (86·9–89·7)	95·2% (95·0–95·4)	1·0%	p=0·06	1·8%	p<0·0001	73·8%
NT@0·97+SN@0·99	88·4% (87·0–89·7)	95·0% (94·9–95·2)	1·2%	p=0·0073	1·7%	p<0·0001	63·0%
NT@0·95+SN@0·97	89·0% (87·7–90·3)	94·5% (94·4–94·7)	1·8%	p=0·0011	1·2%	p<0·0001	73·8%
NT@0·98+SN@0·99	89·3% (87·8–90·6)	94·4% (94·2–94·6)	2·1%	p<0·0001	1·0%	p<0·0001	53·1%
NT@0·99+SN@0·99	89·6% (88·3–91·0)	94·0% (93·8–94·2)	2·4%	p<0·0001	0·6%	p<0·0001	44·5%
NT@0·97+SN@0·98†	89·8% (88·5–91·1)	94·3% (94·2–94·5)	2·6%	p<0·0001	1·0%	p<0·0001	63·0%
NT@0·95+SN@0·95	90·6% (89·3–91·7)	92·9% (92·7–93·1)	3·3%	p<0·0001	−0·4%	p<0·0001	73·8%
NT@0·97+SN@0·97	90·6% (89·4–91·9)	93·7% (93·5–93·9)	3·4%	p<0·0001	0·3%	p=0·0001	63·0%
NT@0·98+SN@0·98	90·7% (89·2–91·9)	93·7% (93·5–93·9)	3·5%	p<0·0001	0·3%	p<0·0001	53·1%
NT@0·99+SN@0·98	91·0% (89·7–92·2)	93·3% (93·1–93·5)	3·8%	p<0·0001	−0·1%	p=0·089	44·5%
NT@0·98+SN@0·97	91·5% (90·3–92·7)	93·0% (92·8–93·2)	4·2%	p<0·0001	−0·3%	p<0·0001	53·1%
NT@0·99+SN@0·97	91·8% (90·6–93·0)	92·6% (92·4–92·8)	4·6%	p<0·0001	−0·8%	p<0·0001	44·5%
NT@0·97+SN@0·95	92·1% (91·0–93·2)	92·1% (91·9–92·3)	4·9%	p<0·0001	−1·3%	p<0·0001	63·0%
NT@0·98+SN@0·95	93·0% (91·9–94·1)	91·4% (91·2–91·6)	5·8%	p<0·0001	−1·9%	p<0·0001	53·1%
NT@0·99+SN@0·95	93·3% (92·2–94·4)	91·0% (90·8–91·2)	6·1%	p<0·0001	−2·4%	p<0·0001	44·5%

Each row represents the operating point achieved on all studies. For decision referral, each row is based on two thresholds that allowed for categorisation of studies going through the decision-referral process into three categories, normal triaging, safety net, and referral to the radiologist. The configuration nomenclature can be understood as NT@ indicating algorithm sensitivity on validation dataset for normal triaging operating point plus SN@ indicating algorithm specificity on validation dataset for safety-net operating point. Threshold setting and selection of operating points on the validation dataset is described in the appendix (p 3). Δ indicates difference in sensitivity and specificity when AI is introduced. NT=normal triaging. SN=safety net.

* Triaging performance is the rate of studies correctly tagged as normal (ie, the fraction of studies that could be automated).

† Exemplary operating point (NT@0·97+SN@0·98).

## Data Availability

Additional information related to this study is available on request to the corresponding author. The code used to process the raw data and to develop the model is tightly integrated with a commercial production system and therefore cannot be released. However, the provenance of the exemplary model used for this work is described in the appendix, which can be used together with open-source deep-learning frameworks such as TensorFlow or PyTorch. The core contribution of this publication is how to use and evaluate any sufficiently accurate model for breast cancer classification on mammograms to pave the way towards clinical applicability. Therefore, all evaluation details are carefully documented and the evaluation part of the code together with data to reproduce figures and tables are available at https://github.com/vara-ai/decision-referral.

## Research in context

### Evidence before this study

We did a PubMed literature search for English-language papers from inception to Sept 1, 2021, to identify studies that used “machine learning”, “deep learning”, “mammography”, and “breast cancer screening” to triage normal exams or detect suspicious lesions on screening mammograms, or which used a decision-referral technique to enhance screening-efficacy parameters via a human-in-the-loop approach, in typical breast-cancer screening populations. We identified artificial intelligence (AI) algorithms for the classification of breast-cancer screening exams that focused on taking over all decisions, including inconclusive cases, not considering downstream effects. In our search we identified AI algorithms that used triaging systems focused on providing negative predictions only, because it has been postulated that such systems could potentially increase the sensitivity of cancer detection by allowing radiologists more time to review serious cases. However, such systems, although greatly reducing the radiologist workload, could also in fact decrease the sensitivity for cancer detection. Other AI approaches are focused on standalone interpretation of screening mammograms to fully automate the detection of suspicious lesions. However, because of the low prevalence of cancers on screening mammograms, such fully automated systems will generate a large number of false positives, requiring more health-care resources to safely triage normal studies. Such approaches could contrarily impair radiologist sensitivity by making excessive positive predictions on benign findings and distract from actual cancers. One published study explored the combination of prescreening and enhanced (supplemental) screening for negative studies with high AI scores; however, this study did not explicitly consider the effect of normal triaging and cancer detection at the point of mammography screening on radiologist sensitivity and specificity.

### Added value of this study

This study aimed to propose a solution toward the safe clinical adoption of AI systems into breast cancer screening. Namely, this study supported adopting a collaborative AI–radiologist approach that combines triage and cancer detection with high accuracy, and forgoes a standalone AI approach that aims to replace the radiologist, but at the risk of degrading sensitivity. Particularly, we propose a decision-referral approach that leverages the strengths of both the radiologist and the AI algorithm. If the algorithm performs more accurately on one subset of studies and the radiologist is better on the other, each can perform predictions in which they excel. This two-part system incorporates both triaging of exams and a safety net to predict cancer-positive exams to maintain a high degree of sensitivity for cancer detection, with the safety net serving as a post-hoc decision support to the radiologist. This approach improves the screening accuracy of radiologists, is adaptive to screening requirements, and allows for the reduction of the workload of radiologists without discarding their final oversight.

### Implications of all the available evidence

The results of this study could improve the safe rollout of AI algorithms, leading to enhanced efficacy parameters in nationwide screening programmes and reduced workloads for radiologists. We show that realistic configurations of our AI algorithm within a decision-referral approach improved screening metrics not only on average but also across clinically relevant subgroups. Furthermore, this approach could be generalised to data from previously unseen screening sites.
