# Supplementary material for: Combining the strengths of radiologists and AI for breast cancer screening: a retrospective analysis
Source: Lancet Digit Health. Author manuscript; Available in PMC 2023 Jan 14. (PMC9839981; doi:10.1016/S2589-7500(22)00070-X)
Supplement: supplemental [file NIHMS1862719-supplement-supplemental.pdf]

THE LANCET
Digital Health

Supplementary appendix

This appendix formed part of the original submission and has been peer reviewed. We post it as supplied by the authors.

Supplement to: Leibig C, Brehmer M, Bunk S, Byng D, Pinker K, Umutlu L. Combining the strengths of radiologists and AI for breast cancer screening: a retrospective analysis. *Lancet Digit Health* 2022; **4:** e507–19.

Online-Only Supplementary Materials accompanying:
Are all clinical decisions equal? A retrospective analysis combining the strengths of radiologists and AI for breast cancer screening

C. Leibig, PhD[*1]; M. Brehmer, MD*[*1,2]; S. Bunk, MSc[1]; D. Byng, MSc[1]; Katja Pinker, MD[†,3,4]; Lale Umutlu, MD[†,2]*
[1]Vara, Berlin, Germany.
[2]Department of Diagnostic and Interventional Radiology and Neuroradiology, University-Hospital Essen, Germany.
[3]Department of Radiology, Breast Imaging Service, Memorial Sloan Kettering Cancer Center, New York, NY, USA.
[4]Department of Biomedical Imaging and Image-guided Therapy Division of Molecular and Gender Imaging, Medical University of Vienna, Vienna, Austria.
*Joint first authors
†Joint last authors

Correspondence:
Name: Christian Leibig
Address: Vara, Max-Urich-Straße 3, 13355 Berlin, Germany
Email: christian.leibig@vara.ai

Contents

eMethods 1. Study inclusion criteria and information regarding the German national breast screening program 2

eMethods 2. Threshold setting and selection of operating points on the validation dataset 3

eMethods 3. Sample weights 6

eMethods 4. Model architecture and training procedure (network training) 8

eTable 1. Accompanying values for Figure 5 (Subgroup sensitivity on external test data for the exemplary operating point NT@97%+SN@98%) 9

eFigure 1. Subgroup sensitivity on internal test data for the exemplary operating point NT@97%+SN@98% 12

eTable 2. Accompanying values for eFigure 1 (Subgroup sensitivity on the internal test data for the exemplary operating point NT@97%+SN@98%) 13

eTable 3. Specificities by manufacturer for stand-alone AI and decision referral (NT@97%+SN@98%) vs. radiologists on the internal test dataset 16

eTable 4. Specificities by manufacturer for stand-alone AI and decision referral (NT@97%+SN@98%) vs. radiologists on the external test dataset 17

eMethods 5. Localization analysis of the safety net 18

References 19

eMethods 1. Study inclusion criteria and information regarding the German national breast screening program

The German national breast screening program operates in full compliance with the quality process indicators of the European guidelines on breast cancer screening (EUREF) and maintains high standards for diagnostic accuracy. In Germany, women are invited to participate in breast screening every two years. A double-reading system is used where initial reads are conducted by two certified physicians who are blinded to each other's decisions. Both readers have professional experience of at least 5,000 mammogram readings per year. Both readers have access to patient records while preparing their assessment, including breast cancer history and images from prior studies. In situations where one or both readers assign BI-RADS > 2, a consensus conference is held whereby a group of readers guided by a leading physician reconciles the differences in interpretation. Each year, ~3% of all women presenting to screening are recalled. 1·1% will undergo biopsy, resulting in 0·59% of the total screening population diagnosed with breast cancer.[1] Recall rates range between 1·4–5·4%, indicating considerable variation between screening sites within one screening system.[1]

The following steps were taken to determine inclusion:

- Confirm the time range (500 days; 27 months) after the initial screening period.
- If there is one malignant biopsy in this period, the study does not count as negative.
- If there is a study in this period, and it does not have a malignant biopsy, then the study is considered a follow-up negative.
- If there is no study in this period (500 days; 27 months), we looked at the next study that followed after the initial screening period, but no later than 4–5 years.

The dates from which the retrospective data were extracted from each screening site are as follows:

Screening Site	Date Range of Extracted Retrospective Data
Internal screening site 1	01 January 2008 – 31 December 2017
Internal screening site 2	01 January 2007 – 26 August 2019
Internal screening site 3	01 January 2007 – 31 December 2018
Internal screening site 4	01 January 2008 – 31 December 2017
Internal screening site 5	01 January 2010 – 30 April 2020
Internal screening site 6	01 January 2008 – 31 December 2017
External screening site 1	01 January 2020 – 31 December 2020
External screening site 2	25 November 2010 – 25 November 2020

The distribution of studies according to ACR breast density categories from the external test dataset (external screening sites 1 and 2) is shown in the bar chart below:

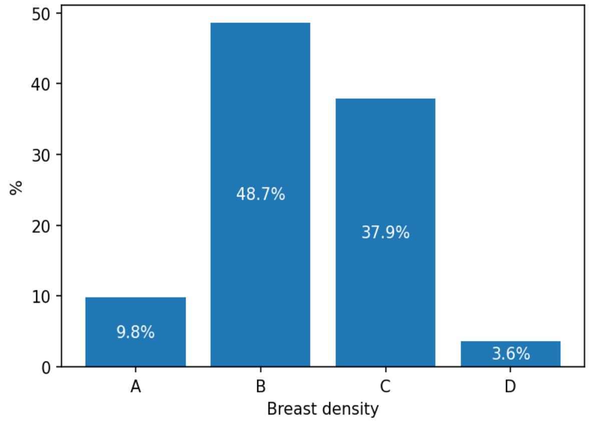

eMethods 2. Threshold setting and selection of operating points on the validation dataset

Here we present the results on the validation dataset (Figure 2), which were used to set all thresholds. For the decision referral approach, the exemplary operating point was determined such that sensitivity is improved maximally without decreasing specificity.

We determined sets of two thresholds which allowed for the categorization of studies going through the decision referral process: i.e., normal triaging, safety net, and referral to the radiologist. Thresholds were represented as sets of two operating points. The nomenclature used in the table below and in Table 1 can be understood as: NT@<*algorithm sensitivity on validation dataset*>+SN@<*algorithm specificity on validation dataset*>.

The stand-alone AI approach required a single threshold to classify studies as positive and negative and was set such that the radiologist's sensitivity was maintained on validation data.

Triaging performance was defined as the rate of studies correctly tagged as normal, i.e., the fraction that could be automated.

Name	Sensitivity (95% CI)	Specificity (95% CI)	Δ Sensitivity (P value)	Δ Specificity (P value)	Triaging performance
Radiologist	86·1% (84·0%, 88·1%)	93·3% (93·0%, 93·6%)			
AI stand-alone	86·1% (84·4%, 87·7%)	88·8% (88·4%, 89·2%)	0·0% (p=1·00)	−4·5% (p<0.0001)	88·8%
NT@0·95+SN@0·99	86·4% (84·6%, 88·3%)	95·3% (95·1%, 95·6%)	0·4% (p=0·61)	2·0% (p<0.0001)	68·5%
NT@0·97+SN@0·99	88·0% (86·0%, 89·7%)	94·5% (94·2%, 94·8%)	1·9% (p=0·0007)	1·2% (p<0.0001)	57·4%
NT@0·95+SN@0·98	88·1% (86·4%, 89·8%)	94·5% (94·2%, 94·8%)	2·1% (p=0·0045)	1·2% (p<0.0001)	68·5%
NT@0·98+SN@0·99	88·6% (86·9%, 90·5%)	93·9% (93·6%, 94·2%)	2·6% (p<0.0001)	0·6% (p<0.0001)	47·0%
NT@0·95+SN@0·97	89·1% (87·4%, 90·8%)	93·7% (93·4%, 94·0%)	3·0% (p<0.0001)	0·4% (p=0·011)	68·5%
NT@0·99+SN@0·99	89·3% (87·5%, 91·0%)	93·5% (93·2%, 93·9%)	3·2% (p<0.0001)	0·2% (p=0·0043)	38·6%
NT@0·97+SN@0·98	89·7% (87·9%, 91·4%)	93·7% (93·4%, 94·0%)	3·6% (p<0.0001)	0·4% (p=0·0011)	57·4%
NT@0·98+SN@0·98	90·3% (88·7%, 91·8%)	93·1% (92·8%, 93·4%)	4·3% (p<0.0001)	−0·2% (p=0·096)	47·0%
NT@0·95+SN@0·95	90·5% (88·9%, 92·2%)	91·9% (91·6%, 92·3%)	4·5% (p<0.0001)	−1·4% (p<0.0001)	68·5%
NT@0·97+SN@0·97	90·6% (89·0%, 92·3%)	92·8% (92·5%, 93·2%)	4·6% (p<0.0001)	−0·5% (p=0·0007)	57·4%
NT@0·99+SN@0·98	91·0% (89·3%, 92·6%)	92·7% (92·4%, 93·0%)	4·9% (p<0.0001)	−0·6% (p<0.0001)	38·6%
NT@0·98+SN@0·97	91·3% (89·7%, 92·8%)	92·3% (91·9%, 92·6%)	5·2% (p<0.0001)	−1·0% (p<0.0001)	47·0%
NT@0·99+SN@0·97	92·0% (90·4%, 93·5%)	91·9% (91·5%, 92·2%)	5·9% (p<0.0001)	−1·4% (p<0.0001)	38·6%
NT@0·97+SN@0·95	92·1% (90·7%, 93·5%)	91·1% (90·7%, 91·5%)	6·0% (p<0.0001)	−2·2% (p<0.0001)	57·4%
NT@0·98+SN@0·95	92·7% (91·3%, 94·1%)	90·5% (90·1%, 90·9%)	6·7% (p<0.0001)	−2·8% (p<0.0001)	47·0%
NT@0·99+SN@0·95	93·4% (92·0%, 94·8%)	90·1% (89·7%, 90·5%)	7·3% (p<0.0001)	−3·2% (p<0.0001)	38·6%

CI: confidence interval, NT: normal triaging, SN: safety net, Δ: difference in sensitivity and specificity when AI is introduced

To further describe the threshold setting, the figures below are presented for illustrative purposes. The model exhibits a score between 0 and 1.0 indicating the malignancy of a study. Scores below the threshold for negative predictions (normal triaging) or above the threshold for positive predictions (safety net) are considered confident; all others, that is, those between the two thresholds are considered unconfident and deferred to the radiologist.

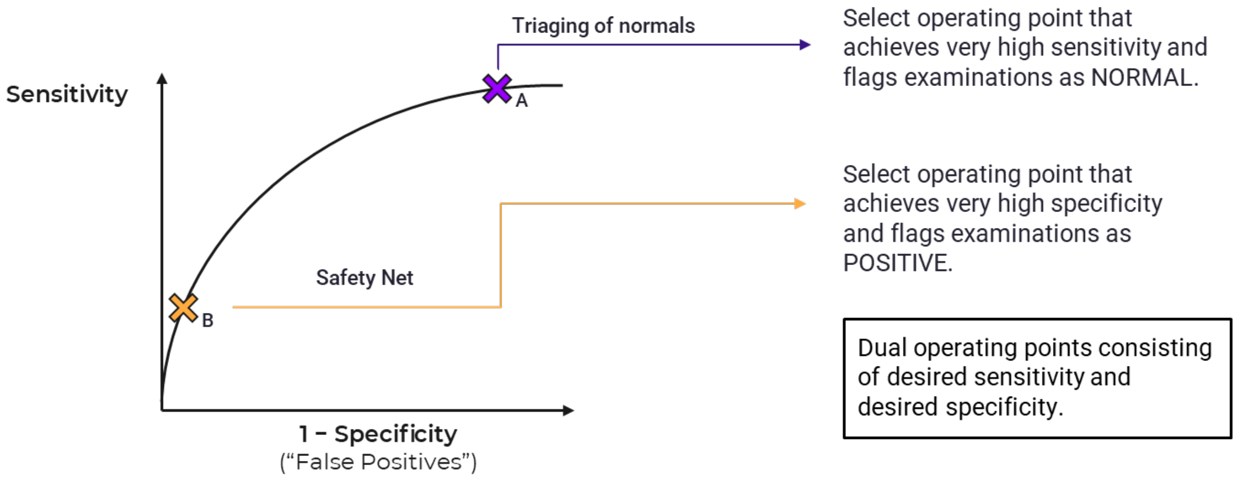

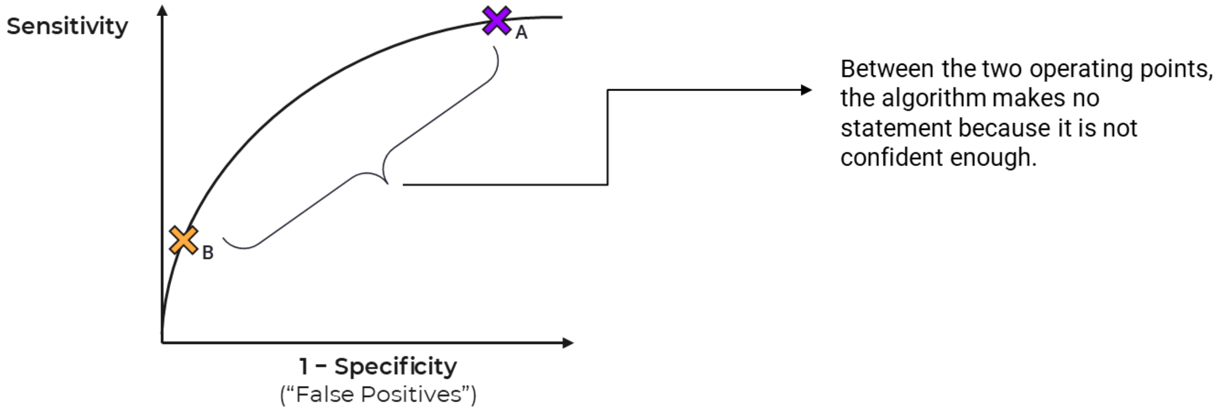

For the stand-alone AI approach, only a single threshold is needed as predictions are performed on all studies. Scores below/above the threshold are considered negative/positive, respectively.

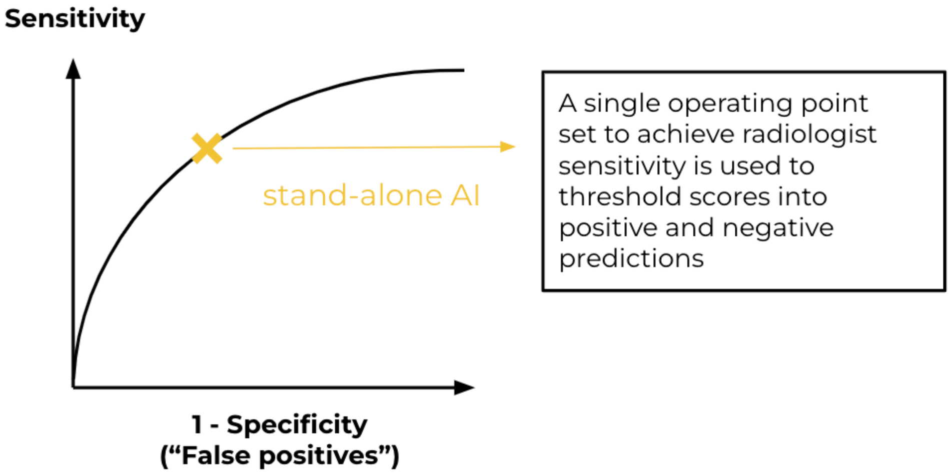

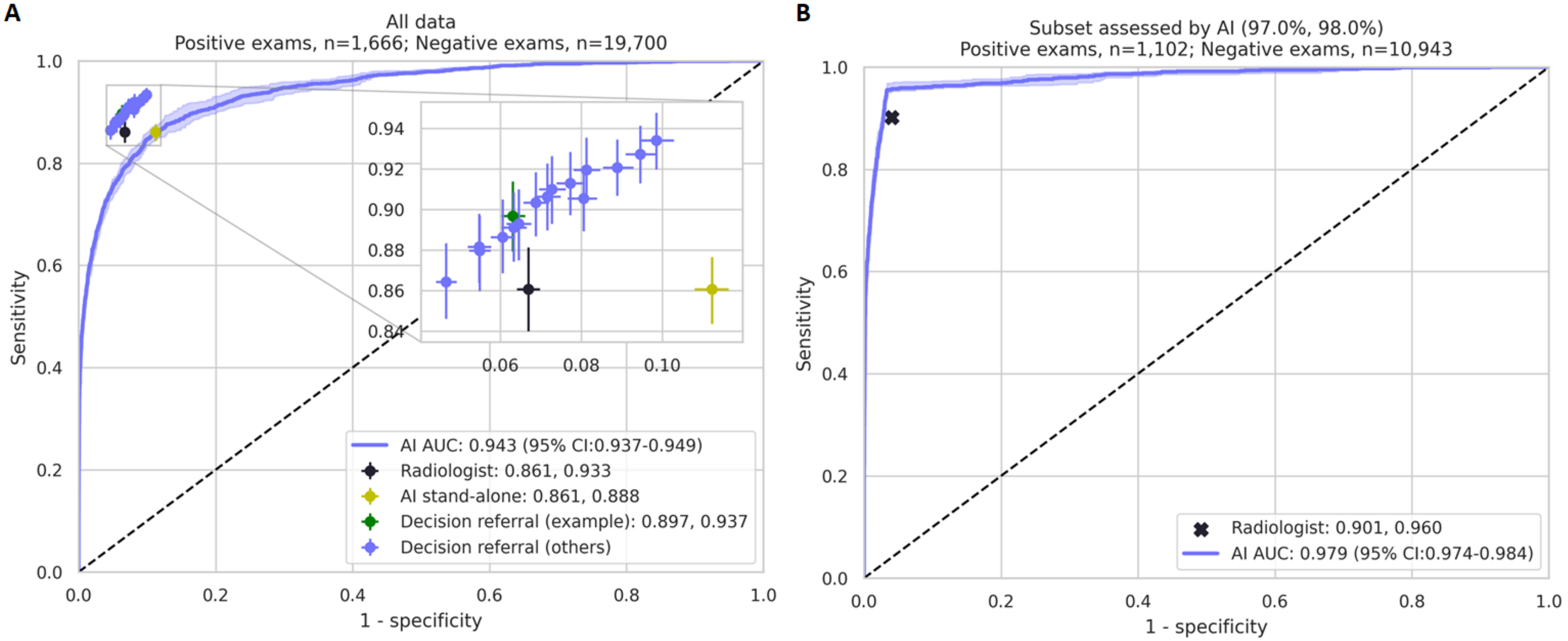

Overall screening diagnostic accuracy for radiologists, stand-alone AI, and decision referral on validation data. Sensitivity and specificity are given for radiologists (black), stand-alone AI (yellow) and decision referral (green for the exemplary configuration NT@97%+SN@98%, blue for alternative configurations from eMethods 2 table). In addition, we present ROC curves and their area under the ROC (AUROC) to evaluate the AI system performance over its entire operating range on the internal validation test dataset (N=21,366) (Figure A) and on the subset of data for which it is able to produce its most confident predictions for the exemplary configuration NT@97%+SN@98% (Figure B). Error bars denote 95% confidence intervals. The decision referral approach outperforms the independent radiologist in either or both sensitivity and specificity depending on the configuration (A) by surpassing the radiologist throughout on the confident set of predictions (B). Resulting sensitivity and specificity values for all studies are comparable to or greater than the radiologist alone, while 38·6%–68·5% of studies are able to be safely triaged (Table 1). AI: artificial intelligence system, AUC: area under the curve, AUROC: area under the receiver operating characteristic curve, CI: confidence interval

eMethods 3. Sample weights

Studies leading to consensus conference, or which were subjected to additional diagnostic imaging and biopsy, make up a small proportion of cases in a real screening population (table below). These are typically oversampled for AI evaluation to accurately estimate the true positive and false positive rate with low variance. To correct for this enrichment in the evaluation and to produce generalizable measures of diagnostic accuracy representative of the actual screening population, inverse probability weighting was used to derive sample weights which reflect the actual distribution of studies in the German breast screening population.[1,3,4] For example, if a certain subset represents 1% of the data, but in the evaluation dataset actually makes up 10%, then each of the individual samples will get a weight of 0·1. Studies assigned to consensus conference and recalled studies are considered ambiguous findings; the percentage of these cases seen in practice likely differ largely by screening sites, and as such, weights must be derived according to screening-specific settings. For the study at hand, we used reference values from the German screening setting. If not stated otherwise, all metrics (ROC curves, specificities, etc.) reported in the main manuscript were computed with inverse probability weighting. To illustrate the impact of reweighting on the performance of the standalone AI system as well as the average radiologist performance, the figure below shows unweighted results in comparison to the internal and external test dataset data shown in Figure 3 and Figure 4 in the main text.

Derivation of sample weights for the external test dataset

	**Percentage in External Test Dataset**	**Actual Percentage in German Screening Population[1]**	**Weight**
**Biopsy-confirmed cancers**	3·37%	0·59%	0·59% / 3·37% = 0·18
**Biopsied but benign**	0·43%	0·51%	0·51% / 0·43% = 1·19
**Recalled but not biopsied**	3·17%	1·80%	1·80% / 3·17% = 0·57
**Consensus conference but no recall**	7·82%	9·30%	9·30% / 7·82% = 1·19
**No consensus conference**	85·22%	87·80%	87·80% / 85·22% = 1·02

Unweighted performance results on the external test dataset

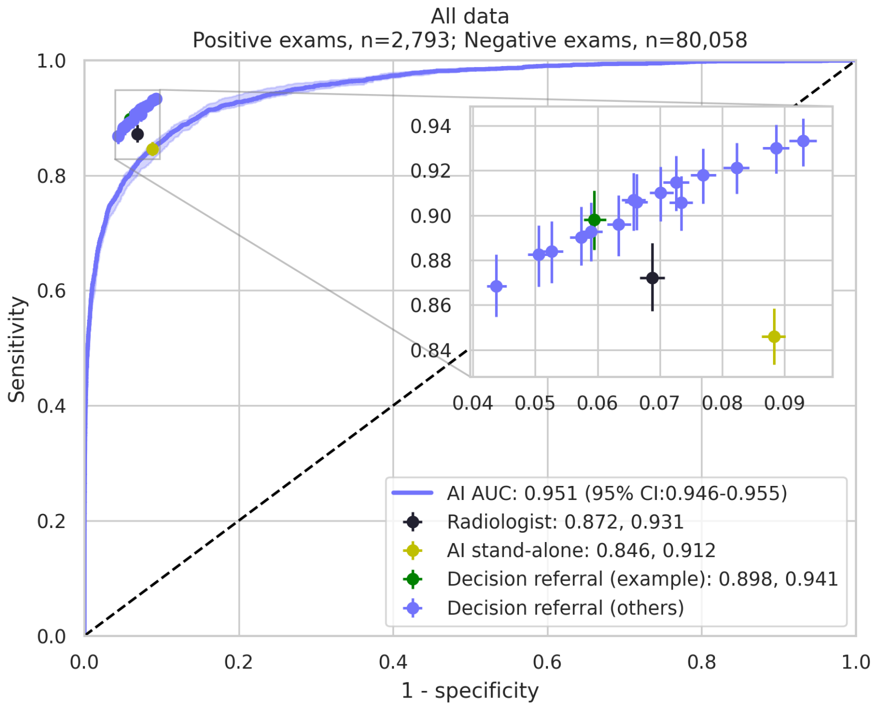

Derivation of sample weights for the internal test dataset

	Percentage in Internal Test Dataset	Actual Percentage in German Screening Population[1]	Weight
**Biopsy-confirmed cancers**	7·71%	0·59%	0·59% / 7·71% = 0·08
**Biopsied but benign**	1·02%	0·51%	0·51% / 1·02% = 0·50
**Recalled but not biopsied**	6·91%	1·80%	1·80% / 6·91% = 0·26
**Consensus conference but no recall**	15·28%	9·30%	9·30% / 15·28% = 0·61
**No consensus conference**	69·08%	87·80%	87·80% / 69·08% = 1·26

Unweighted performance results on internal test dataset

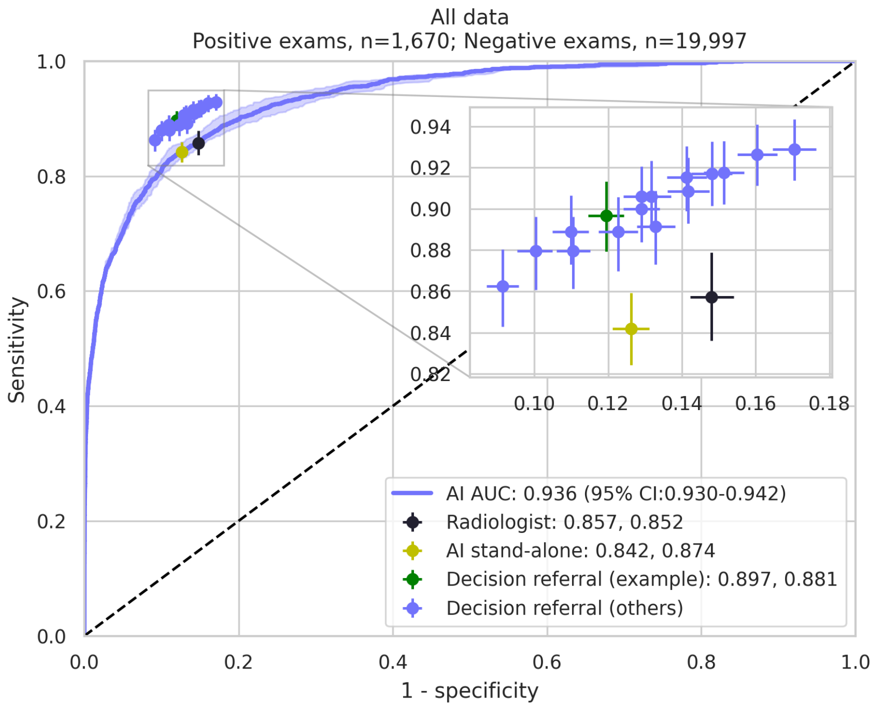

eMethods 4. Model architecture and training procedure (network training)

The main component of the model is a deep CNN with 34 layers. An ensemble of multiple such models was used to mitigate overconfident predictions under dataset shift.[2] The strength of CNNs to learn hierarchical feature representations was also considered for the design of the three-step training strategy: (1) a smaller network was pre-trained to classify patches in order to capture the fine-grained, texture like information of different lesion types and their malignancy. (2) The pre-trained network from (1) served as initialization of a larger network that was trained to perform cancer classification on an image level. This requires the network to learn how to aggregate local lesion into global image information. (3) A logistic regression model was trained to perform study-level cancer classification, aggregating information from the different image classifications in the study. Separate study models were used for the safety net feature and triaging in order to optimize both specificity and sensitivity.

The outputs of the study-level models are real values [0·0 to 1·0]. These are converted to binary decisions by applying a threshold. For the triaging model, a threshold is determined based on the desired sensitivity. All predictions below that threshold are considered negative. For the safety net model, a threshold is determined based on the desired specificity. All predictions above that threshold are considered positive. Predictions that are considered neither positive nor negative are referred to the radiologist. An illustration of this threshold setting is given in eMethods 2.

Patches centered around malignant and benign lesion annotations were used for pre-training a CNN with 28 layers, whose weights were subsequently used to initialize a deeper network with 34 layers to learn the aggregation of patch level to image level features. At the patch level, a multi-task loss minimized the cross-entropy between output units and lesion malignancy (ground truth label from histopathology) as well as the associated radiological findings (using annotations according to BI-RADS as described in the main manuscript). Optimization was performed via stochastic gradient descent (learning rate=1e-4, momentum=0·9) for 60k steps and a batch size of 100. Early stopping based on the validation loss was applied to select the set of weights to be used for initializing the image model. Image-level malignancy labels were derived from associated radiology and histopathology reports. Image-level labels were learned via cross-entropy minimization running SGD for 60k steps with a batch size of 36 (maximized to fit into the memory of 4 GPUs) and a cosine-decayed learning rate of 2e-3. Early stopping based on the image level validation loss was used to determine the final set of weights. This set of weights was used to extract features for the study level logistic regression model. Besides early stopping, overfitting was prevented via L2 regularization and data augmentation applied to both patches and images (translation, rotation, flipping, and fractional rescaling). Class balancing was applied during both patch- as well as image-level training. Four different study-level models were trained using slightly different hyperparameter settings in order to encourage prediction diversity. The ensemble member scores were averaged in order to get a single prediction per study.

eTable 1. Accompanying values for Figure 5 (Subgroup sensitivity on external test data for the exemplary operating point NT@97%+SN@98%)

Method	Stratification	Stratum	No. studies	Sensitivity (95% CI)	Δ Sensitivity (P value)
Rad.			2793	87·2% (85·6%, 88·7%)	
AI			2793	84·6% (83·3%, 86·0%)	−2·6% (p=0·0009)
DR			2793	89·8% (88·5%, 91·1%)	2·6% (p=0)
Rad.	Screening site	External site 1	522	88·2% (84·5%, 91·6%)	
AI	Screening site	External site 1	522	87·0% (84·1%, 89·7%)	−1·2% (p=0·49)
DR	Screening site	External site 1	522	91·2% (88·1%, 93·9%)	3·0% (p=0·0092)
Rad.	Screening suite	External site 2	2271	87·0% (85·2%, 88·6%)	
AI	Screening site	External site 2	2271	84·1% (82·6%, 85·5%)	−2·9% (p=0·0019)
DR	Screening site	External site 2	2271	89·5% (88·0%, 91·0%)	2·5% (p=0·0001)
Rad.	Manufacturer	Fuji	289	87·5% (82·7%, 92·4%)	
AI	Manufacturer	Fuji	289	86·5% (82·7%, 90·0%)	−1·0% (p=0·7)
DR	Manufacturer	Fuji	289	91·9% (87·9%, 95·5%)	4·3% (p=0·0035)
Rad.	Manufacturer	Hologic	680	87·8% (84·7%, 90·7%)	
AI	Manufacturer	Hologic	680	87·9% (85·6%, 90·3%)	0·1% (p=0·94)
DR	Manufacturer	Hologic	680	91·0% (88·5%, 93·1%)	3·2% (p=0·0034)
Rad.	Manufacturer	Siemens	1824	87·0% (85·1%, 88·9%)	
AI	Manufacturer	Siemens	1824	83·1% (81·3%, 84·7%)	−3·9% (p=0·0002)
DR	Manufacturer	Siemens	1824	89·0% (87·3%, 90·7%)	2·1% (p=0·0002)
Rad.	Breast density	ACR-A	112	87·9% (80·4%, 94·7%)	
AI	Breast density	ACR-A	112	85·7% (78·6%, 92·0%)	−2·2% (p=0·58)
DR	Breast density	ACR-A	112	92·4% (85·7%, 97·3%)	4·5% (p=0·06)
Rad.	Breast density	ACR-B	1673	88·1% (86·3%, 90·0%)	
AI	Breast density	ACR-B	1673	85·2% (83·6%, 87·0%)	−2·8% (p=0·0066)
DR	Breast density	ACR-B	1673	90·7% (89·0%, 92·2%)	2·6% (p=0·0001)
Rad.	Breast density	ACR-C	951	85·5% (82·8%, 88·2%)	
AI	Breast density	ACR-C	951	83·7% (81·5%, 86·0%)	−1·8% (p=0·24)
DR	Breast density	ACR-C	951	87·9% (85·5%, 90·1%)	2·4% (p=0·0045)
Rad.	Breast density	ACR-D	55	89·1% (78·1%, 98·2%)	
AI	Breast density	ACR-D	55	80·0% (69·1%, 89·1%)	−9·1% (p=0·13)
DR	Breast density	ACR-D	55	90·9% (81·8%, 98·2%)	1·8% (p=0·64)
Rad.	Size (mm)	T1 (≤20)	1385	84·1% (81·7%, 86·5%)	
AI	Size (mm)	T1 (≤20)	1385	81·9% (79·7%, 84·0%)	−2·2% (p=0·089)
DR	Size (mm)	T1 (≤20)	1385	87·3% (85·2%, 89·3%)	3·2% (p=0)
Rad.	Size (mm)	T2 (20-50)	1077	91·3% (89·1%, 93·2%)	
AI	Size (mm)	T2 (20-50)	1077	87·8% (85·8%, 89·6%)	−3·5% (p=0·0032)
DR	Size (mm)	T2 (20-50)	1077	92·9% (91·1%, 94·5%)	1·6% (p=0·029)

Rad.	Size (mm)	T3 (>50)	89	93·3% (86·5%, 98·9%)	
AI	Size (mm)	T3 (>50)	89	93·3% (87·6%, 97·8%)	0·0% (p=1)
DR	Size (mm)	T3 (>50)	89	96·6% (91·0%, 100·0%)	3·4% (p=0·14)
Rad.	Core Needle Biopsy score	B3 - Uncertain malignant potential	340	77·4% (71·2%, 82·9%)	
AI	Core Needle Biopsy score	B3 - Uncertain malignant potential	340	70·0% (65·3%, 74·7%)	−7·4% (p=0·018)
DR	Core Needle Biopsy score	B3 - Uncertain malignant potential	340	78·2% (72·4%, 83·2%)	0·9% (p=0·63)
Rad.	Core Needle Biopsy score	B4 - Suspicious	42	82·1% (66·7%, 95·2%)	
AI	Core Needle Biopsy score	B4 - Suspicious	42	76·2% (64·3%, 88·1%)	−6·0% (p=0·46)
DR	Core Needle Biopsy score	B4 - Suspicious	42	84·5% (69·0%, 95·2%)	2·4% (p=0·69)
Rad.	Core Needle Biopsy score	B5a - Malignant (In situ)	465	85·7% (81·7%, 89·5%)	
AI	Core Needle Biopsy score	B5a - Malignant (In situ)	465	91·2% (88·8%, 93·8%)	5·5% (p=0·0036)
DR	Core Needle Biopsy score	B5a - Malignant (In situ)	465	90·6% (87·5%, 93·8%)	4·9% (p=0·0001)
Rad.	Core Needle Biopsy score	B5b - Malignant (Invasive)	1923	89·4% (87·6%, 91·2%)	
AI	Core Needle Biopsy score	B5b - Malignant (Invasive)	1923	86·0% (84·5%, 87·4%)	−3·4% (p=0·0006)
DR	Core Needle Biopsy score	B5b - Malignant (Invasive)	1923	92·0% (90·5%, 93·4%)	2·5% (p=0)
Rad.	Finding	Architectural distortion	358	83·4% (78·5%, 88·0%)	
AI	Finding	Architectural distortion	358	75·7% (70·9%, 79·9%)	−7·7% (p=0·0036)
DR	Finding	Architectural distortion	358	86·5% (82·1%, 90·2%)	3·1% (p=0·069)
Rad.	Finding	Asymmetry	230	88·7% (83·5%, 93·5%)	
AI	Finding	Asymmetry	230	79·6% (74·3%, 84·8%)	−9·1% (p=0·0019)
DR	Finding	Asymmetry	230	87·4% (82·2%, 92·2%)	−1·3% (p=0·5)
Rad.	Finding	Axillary lymph node	64	92·2% (82·8%, 98·4%)	
AI	Finding	Axillary lymph node	64	82·8% (71·9%, 90·6%)	−9·4% (p=0·054)
DR	Finding	Axillary lymph node	64	86·7% (78·1%, 93·7%)	−5·5% (p=0·097)
Rad.	Finding	Calcification	1025	86·6% (84·0%, 89·1%)	
AI	Finding	Calcification	1025	90·5% (88·7%, 92·3%)	3·9% (p=0·0014)
DR	Finding	Calcification	1025	91·0% (88·8%, 93·2%)	4·4% (p=0)
Rad.	Finding	Global asymmetry	4	100·0% (100·0%, 100·0%)	
AI	Finding	Global asymmetry	4	75·0% (25·0%, 100·0%)	−25·0% (p=1)
DR	Finding	Global asymmetry	4	75·0% (25·0%, 100·0%)	−25·0% (p=1)
Rad.	Finding	Intramammary lymph node	435	86·8% (82·8%, 90·6%)	
AI	Finding	Intramammary lymph node	435	86·4% (83·2%, 89·7%)	−0·3% (p=0·87)
DR	Finding	Intramammary lymph node	435	89·9% (86·7%, 93·1%)	3·1% (p=0·012)

Rad.	Finding	Mass	1511	89·9% (87·9%, 91·7%)	
AI	Finding	Mass	1511	86·1% (84·3%, 88·0%)	−3·8% (p=0)
DR	Finding	Mass	1511	91·9% (90·2%, 93·3%)	1·9% (p=0·0013)
Rad.	Finding	Nipple retraction	20	90·0% (70·0%, 100·0%)	
AI	Finding	Nipple retraction	20	90·0% (75·0%, 100·0%)	0·0% (p=1)
DR	Finding	Nipple retraction	20	92·5% (75·0%, 100·0%)	2·5% (p=0·49)
Rad.	Finding	Skin lesion	68	89·7% (80·9%, 97·1%)	
AI	Finding	Skin lesion	68	91·2% (83·8%, 97·1%)	1·5% (p=0·66)
DR	Finding	Skin lesion	68	94·9% (88·2%, 100·0%)	5·1% (p=0·098)
Rad.	Finding	Skin retraction	17	91·2% (70·6%, 100·0%)	
AI	Finding	Skin retraction	17	82·4% (64·7%, 100·0%)	−8·8% (p=0·44)
DR	Finding	Skin retraction	17	91·2% (76·5%, 100·0%)	0·0% (p=1)
Rad.	Finding	Skin thickening	5	100·0% (100·0%, 100·0%)	
AI	Finding	Skin thickening	5	80·0% (40·0%, 100·0%)	−20·0% (p=1)
DR	Finding	Skin thickening	5	100·0% (100·0%, 100·0%)	0·0% (p=1)
Rad.	Finding	Solitary dilated duct	6	100·0% (100·0%, 100·0%)	
AI	Finding	Solitary dilated duct	6	100·0% (100·0%, 100·0%)	0·0% (p=1)
DR	Finding	Solitary dilated duct	6	100·0% (100·0%, 100·0%)	0·0% (p=1)

AI: artificial intelligence system, DR: Decision referral approach, Rad.: radiologist

eFigure 1. Subgroup sensitivity on internal test data for the exemplary operating point NT@97%+SN@98%

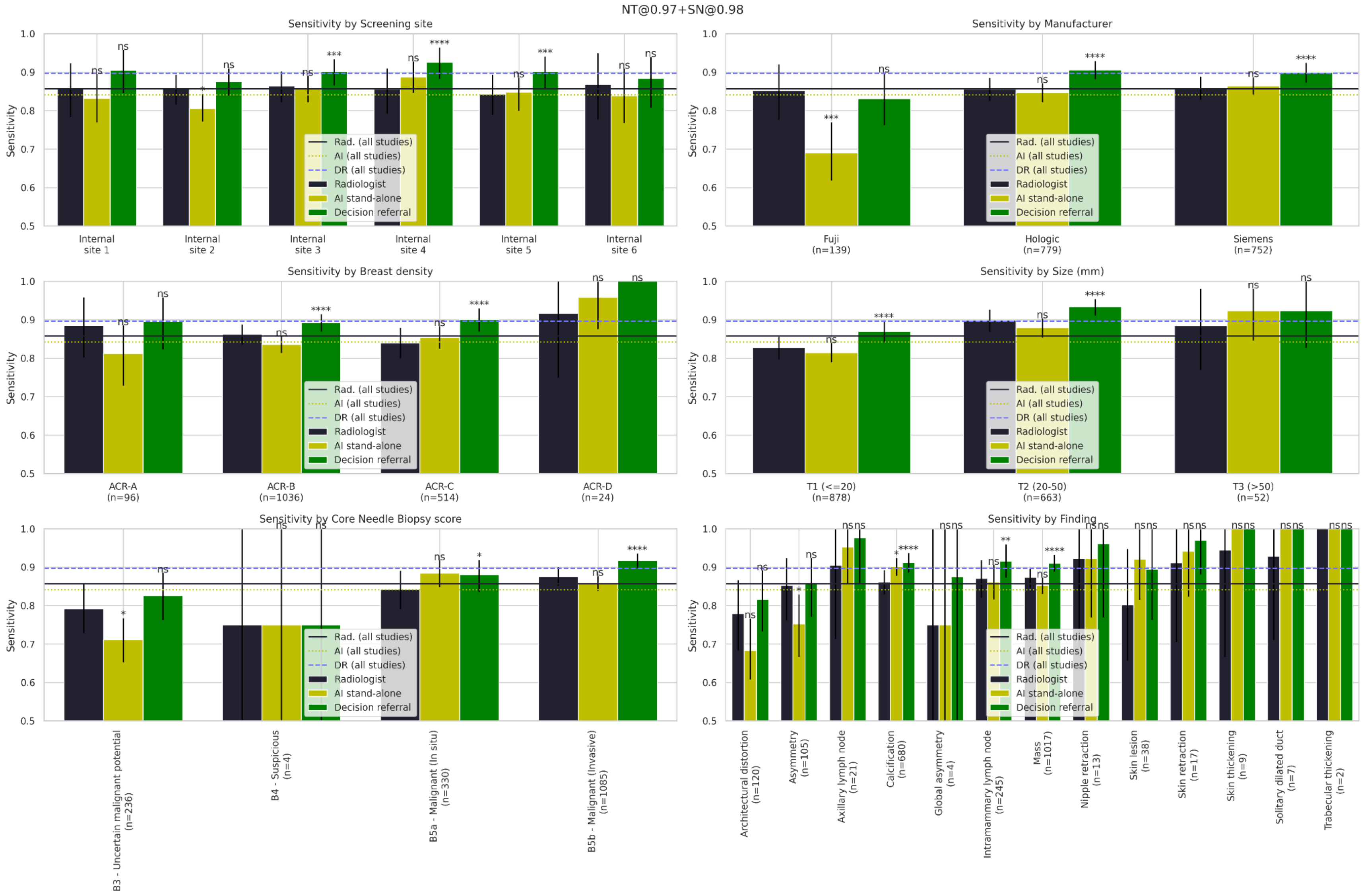

eTable 2. Accompanying values for eFigure 1 (Subgroup sensitivity on the internal test data for the exemplary operating point NT@97%+SN@98%)

Method	Stratification	Stratum	No. studies	Sensitivity (95% CI)	Δ Sensitivity (P value)
Rad.			1670	85·7% (83·6%, 87·8%)	
AI			1670	84·2% (82·5%, 85·9%)	-1·5% (p=0·17)
DR			1670	89·7% (88·0%, 91·3%)	4·0% (p=0)
Rad.	Screening site	Internal site 1	143	85·7% (78·3%, 92·3%)	
AI	Screening site	Internal site 1	143	83·2% (76·9%, 89·5%)	−2·4% (p=0·54)
DR	Screening site	Internal site 1	143	90·6% (84·6%, 95·8%)	4·9% (p=0·083)
Rad.	Screening site	Internal site 2	478	85·8% (81·6%, 89·3%)	
AI	Screening site	Internal site 2	478	80·5% (77·2%, 84·3%)	−5·2% (p=0·017)
DR	Screening site	Internal site 2	478	87·6% (83·9%, 91·0%)	1·8% (p=0·15)
Rad.	Screening site	Internal site 3	438	86·4% (82·2%, 90·2%)	
AI	Screening site	Internal site 3	438	85·8% (82·2%, 89·0%)	−0·6% (p=0·78)
DR	Screening site	Internal site 3	438	90·2% (86·5%, 93·4%)	3·8% (p=0·0007)
Rad.	Screening site	Internal site 4	222	85·6% (79·3%, 91·0%)	
AI	Screening site	Internal site 4	222	88·7% (84·7%, 92·8%)	3·2% (p=0·33)
DR	Screening site	Internal site 4	222	92·6% (88·3%, 96·4%)	7·0% (p=0·0001)
Rad.	Screening site	Internal site 5	290	84·3% (79·0%, 89·3%)	
AI	Screening site	Internal site 5	290	84·8% (80·0%, 88·6%)	0·5% (p=0·85)
DR	Screening site	Internal site 5	290	90·2% (85·5%, 94·1%)	5·9% (p=0·0004)
Rad.	Screening site	Internal site 6	99	86·9% (77·8%, 94·9%)	
AI	Screening site	Internal site 6	99	83·8% (76·7%, 90·9%)	−3·0% (p=0·57)
DR	Screening site	Internal site 6	99	88·4% (80·8%, 93·9%)	1·5% (p=0·67)
Rad.	Manufacturer	Fuji	139	85·3% (77·7%, 92·1%)	
AI	Manufacturer	Fuji	139	69·1% (61·9%, 77·0%)	−16·2% (p=0·0009)
DR	Manufacturer	Fuji	139	83·1% (76·3%, 89·9%)	−2·2% (p=0·4)
Rad.	Manufacturer	Hologic	779	85·6% (82·5%, 88·6%)	
AI	Manufacturer	Hologic	779	84·7% (82·2%, 87·2%)	−0·8% (p=0·61)
DR	Manufacturer	Hologic	779	90·6% (88·2%, 92·9%)	5·0% (p=0)
Rad.	Manufacturer	Siemens	752	86·0% (82·8%, 88·8%)	
AI	Manufacturer	Siemens	752	86·4% (84·0%, 88·7%)	0·5% (p=0·77)
DR	Manufacturer	Siemens	752	90·0% (87·4%, 92·4%)	4·0% (p=0)
Rad.	Breast density	ACR-A	96	88·5% (80·2%, 95·8%)	
AI	Breast density	ACR-A	96	81·2% (72·9%, 88·5%)	−7·3% (p=0·092)
DR	Breast density	ACR-A	96	89·6% (82·3%, 95·8%)	1·0% (p=0·68)
Rad.	Breast density	ACR-B	1036	86·2% (83·5%, 88·7%)	
AI	Breast density	ACR-B	1036	83·6% (81·4%, 85·7%)	−2·6% (p=0·069)
DR	Breast density	ACR-B	1036	89·2% (87·0%, 91·4%)	3·0% (p=0)

Rad.	Breast density	ACR-C	514	83·9% (80·0%, 87·9%)	
AI	Breast density	ACR-C	514	85·4% (82·5%, 88·3%)	1·5% (p=0·48)
DR	Breast density	ACR-C	514	90·1% (87·0%, 93·0%)	6·1% (p=0)
Rad.	Breast density	ACR-D	24	91·7% (75·0%, 100·0%)	
AI	Breast density	ACR-D	24	95·8% (87·5%, 100·0%)	4·2% (p=0·69)
DR	Breast density	ACR-D	24	100·0% (100·0%, 100·0%)	8·3% (p=0·29)
Rad.	Size (mm)	T1 (≤20)	878	82·7% (79·7%, 85·5%)	
AI	Size (mm)	T1 (≤20)	878	81·4% (78·9%, 83·9%)	−1·3% (p=0·44)
DR	Size (mm)	T1 (≤20)	878	87·0% (84·1%, 89·6%)	4·3% (p=0)
Rad.	Size (mm)	T2 (20-50)	663	89·9% (86·9%, 92·6%)	
AI	Size (mm)	T2 (20-50)	663	87·9% (85·4%, 90·3%)	−2·0% (p=0·22)
DR	Size (mm)	T2 (20-50)	663	93·4% (91·1%, 95·3%)	3·5% (p=0)
Rad.	Size (mm)	T3 (>50)	52	88·5% (76·9%, 98·1%)	
AI	Size (mm)	T3 (>50)	52	92·3% (84·6%, 98·1%)	3·8% (p=0·52)
DR	Size (mm)	T3 (>50)	52	92·3% (82·7%, 100·0%)	3·8% (p=0·33)
Rad.	Core Needle Biopsy score	B3 - Uncertain malignant potential	236	79·2% (72·9%, 85·6%)	
AI	Core Needle Biopsy score	B3 - Uncertain malignant potential	236	71·2% (65·3%, 76·7%)	−8·1% (p=0·031)
DR	Core Needle Biopsy score	B3 - Uncertain malignant potential	236	82·6% (76·3%, 88·6%)	3·4% (p=0·1)
Rad.	Core Needle Biopsy score	B4 - Suspicious	4	75·0% (25·0%, 100·0%)	
AI	Core Needle Biopsy score	B4 - Suspicious	4	75·0% (25·0%, 100·0%)	0·0% (p=1)
DR	Core Needle Biopsy score	B4 - Suspicious	4	75·0% (25·0%, 100·0%)	0·0% (p=1)
Rad.	Core Needle Biopsy score	B5a - Malignant (In situ)	330	84·2% (79·1%, 89·1%)	
AI	Core Needle Biopsy score	B5a - Malignant (In situ)	330	88·5% (84·8%, 91·8%)	4·2% (p=0·095)
DR	Core Needle Biopsy score	B5a - Malignant (In situ)	330	88·0% (83·6%, 91·8%)	3·8% (p=0·01)
Rad.	Core Needle Biopsy score	B5b - Malignant (Invasive)	1085	87·6% (85·2%, 90·0%)	
AI	Core Needle Biopsy score	B5b - Malignant (Invasive)	1085	85·7% (83·8%, 87·7%)	−1·9% (p=0·14)
DR	Core Needle Biopsy score	B5b - Malignant (Invasive)	1085	91·8% (89·8%, 93·5%)	4·1% (p=0)
Rad.	Finding	Architectural distortion	120	77·9% (68·3%, 86·7%)	
AI	Finding	Architectural distortion	120	68·3% (60·8%, 76·7%)	−9·6% (p=0·051)
DR	Finding	Architectural distortion	120	81·7% (73·3%, 89·2%)	3·7% (p=0·21)
Rad.	Finding	Asymmetry	105	85·2% (76·2%, 92·4%)	

AI	Finding	Asymmetry	105	75·2% (66·7%, 82·9%)	−10·0% (p=0·045)
DR	Finding	Asymmetry	105	85·7% (77·1%, 92·4%)	0·5% (p=0·86)
Rad.	Finding	Axillary lymph node	21	90·5% (71·4%, 100·0%)	
AI	Finding	Axillary lymph node	21	95·2% (85·7%, 100·0%)	4·8% (p=0·72)
DR	Finding	Axillary lymph node	21	97·6% (85·7%, 100·0%)	7·1% (p=0·22)
Rad.	Finding	Calcification	680	86·1% (82·9%, 89·3%)	
AI	Finding	Calcification	680	90·1% (87·8%, 92·4%)	4·0% (p=0·015)
DR	Finding	Calcification	680	91·2% (88·7%, 93·7%)	5·1% (p=0)
Rad.	Finding	Global asymmetry	4	75·0% (25·0%, 100·0%)	
AI	Finding	Global asymmetry	4	75·0% (25·0%, 100·0%)	0·0% (p=1)
DR	Finding	Global asymmetry	4	87·5% (50·0%, 100·0%)	12·5% (p=0·5)
Rad.	Finding	Intramammary lymph node	245	87·1% (82·0%, 91·8%)	
AI	Finding	Intramammary lymph node	245	86·1% (81·6%, 90·6%)	−1·0% (p=0·69)
DR	Finding	Intramammary lymph node	245	91·6% (87·3%, 95·9%)	4·5% (p=0·0086)
Rad.	Finding	Mass	1017	87·4% (84·8%, 89·9%)	
AI	Finding	Mass	1017	85·3% (83·1%, 87·3%)	−2·2% (p=0·12)
DR	Finding	Mass	1017	91·1% (88·9%, 93·2%)	3·7% (p=0)
Rad.	Finding	Nipple retraction	13	92·3% (69·2%, 100·0%)	
AI	Finding	Nipple retraction	13	92·3% (76·9%, 100·0%)	0·0% (p=1)
DR	Finding	Nipple retraction	13	96·2% (76·9%, 100·0%)	3·8% (p=0·51)
Rad.	Finding	Skin lesion	38	80·3% (65·7%, 94·7%)	
AI	Finding	Skin lesion	38	92·1% (81·6%, 100·0%)	11·8% (p=0·16)
DR	Finding	Skin lesion	38	89·5% (76·3%, 100·0%)	9·2% (p=0·058)
Rad.	Finding	Skin retraction	17	91·2% (70·6%, 100·0%)	
AI	Finding	Skin retraction	17	94·1% (82·4%, 100·0%)	2·9% (p=0·68)
DR	Finding	Skin retraction	17	97·1% (88·1%, 100·0%)	5·9% (p=0·13)
Rad.	Finding	Skin thickening	9	94·4% (66·7%, 100·0%)	
AI	Finding	Skin thickening	9	100·0% (100·0%, 100·0%)	5·6% (p=0·51)
DR	Finding	Skin thickening	9	100·0% (100·0%, 100·0%)	5·6% (p=0·51)
Rad.	Finding	Solitary dilated duct	7	92·9% (71·1%, 100·0%)	
AI	Finding	Solitary dilated duct	7	100·0% (100·0%, 100·0%)	7·1% (p=0·5)
DR	Finding	Solitary dilated duct	7	100·0% (100·0%, 100·0%)	7·1% (p=0·5)
Rad.	Finding	Trabecular thickening	2	100·0% (100·0%, 100·0%)	
AI	Finding	Trabecular thickening	2	100·0% (100·0%, 100·0%)	0·0% (p=1)
DR	Finding	Trabecular thickening	2	100·0% (100·0%, 100·0%)	0·0% (p=1)

AI: artificial intelligence system, DR: Decision referral approach, Rad.: radiologist

eTable 3. Specificities by manufacturer for stand-alone AI and decision referral (NT@97%+SN@98%) vs. radiologists on the internal test dataset

Method	Stratification	Stratum	No. studies	Specificity (95% CI)	Δ Specificity (P value)
Rad.			19997	93.4% (93.1%, 93.7%)	
AI			19997	89.5% (89.0%, 89.9%)	−3.9% (p=0)
DR			19997	93.8% (93.6%, 94.1%)	0.5% (p=0.0002)
Rad.	Manufacturer	Fuji	1379	93.3% (92.1%, 94.3%)	
AI	Manufacturer	Fuji	1379	95.7% (94.7%, 96.8%)	2.4% (p=0.0004)
DR	Manufacturer	Fuji	1379	95.9% (94.9%, 96.7%)	2.6% (p=0)
Rad.	Manufacturer	Hologic	10643	93.5% (93.1%, 93.8%)	
AI	Manufacturer	Hologic	10643	88.7% (88.1%, 89.3%)	−4.7% (p=0)
DR	Manufacturer	Hologic	10643	93.6% (93.2%, 94.1%)	0.2% (p=0.28)
Rad.	Manufacturer	Siemens	7975	93.3% (92.8%, 93.8%)	
AI	Manufacturer	Siemens	7975	89.4% (88.6%, 90.0%)	−3.9% (p=0)
DR	Manufacturer	Siemens	7975	93.8% (93.3%, 94.2%)	0.5% (p=0.009)

AI: artificial intelligence system, DR: Decision referral approach, Rad.: radiologist

eTable 4. Specificities by manufacturer for stand-alone AI and decision referral (NT@97%+SN@98%) vs. radiologists on the external test dataset

Method	Stratification	Stratum	No. studies	Specificity (95% CI)	Δ Specificity (P value)
Rad.			80058	93.4% (93.2%, 93.6%)	
AI			80058	91.3% (91.1%, 91.5%)	−2.0% (p=0)
DR			80058	94.3% (94.2%, 94.5%)	1.0% (p=0)
Rad.	Manufacturer	Fuji	15653	95.0% (94.6%, 95.4%)	
AI	Manufacturer	Fuji	15653	93.1% (92.6%, 93.4%)	−2.0% (p=0)
DR	Manufacturer	Fuji	15653	95.8% (95.4%, 96.1%)	0.8% (p=0)
Rad.	Manufacturer	Hologic	19121	93.4% (92.9%, 93.8%)	
AI	Manufacturer	Hologic	19121	88.9% (88.4%, 89.3%)	−4.5% (p=0)
DR	Manufacturer	Hologic	19121	93.3% (92.9%, 93.7%)	-0.1% (p=0.49)
Rad.	Manufacturer	Siemens	45284	92.8% (92.5%, 93.1%)	
AI	Manufacturer	Siemens	45284	91.8% (91.5%, 92.0%)	−1.0% (p=0)
DR	Manufacturer	Siemens	45284	94.3% (94.0%, 94.5%)	1.5% (p=0)

AI: artificial intelligence system, DR: Decision referral approach, Rad.: radiologist

eMethods 5. Localization analysis of the safety net

The safety net's predictions would in practice be shown to the user. In order to localize the lesion deemed most suspicious by the safety net, we explained those images that would trigger the safety net by computing approximate SHAP values for the network layer that corresponds to the patch level classifications (training stage 1).[5] This gives us x-y coordinates pointing to lesions in images.

In the following, we analyzed how well the safety net's marker positions (x, y coordinates in the image) can localize biopsy-proven, malignant lesions by checking whether the x,y coordinates would fall inside the rectangular bounding box surrounding polygon annotations. Each image can individually cause the safety net to be triggered and we display the marker position on each of those images. For that, we filter the dataset to images that contain at least one malignant annotation. This allows us to analyze the localization performance on an image level: if the marker position resides inside any malignant annotation, we count this as a hit. The figure displays the localization performance as a fraction over all images vs. specificity (x-axis, i.e. different operating points). There is an important trend: the more confident (higher specificity) the model predictions, the better the localization performance. Exemplarily, for a specificity of 98·0%, we could correctly localize ~95% of the findings. We believe that this will support the radiologist in detecting the most suspicious lesions flagged by the safety net.

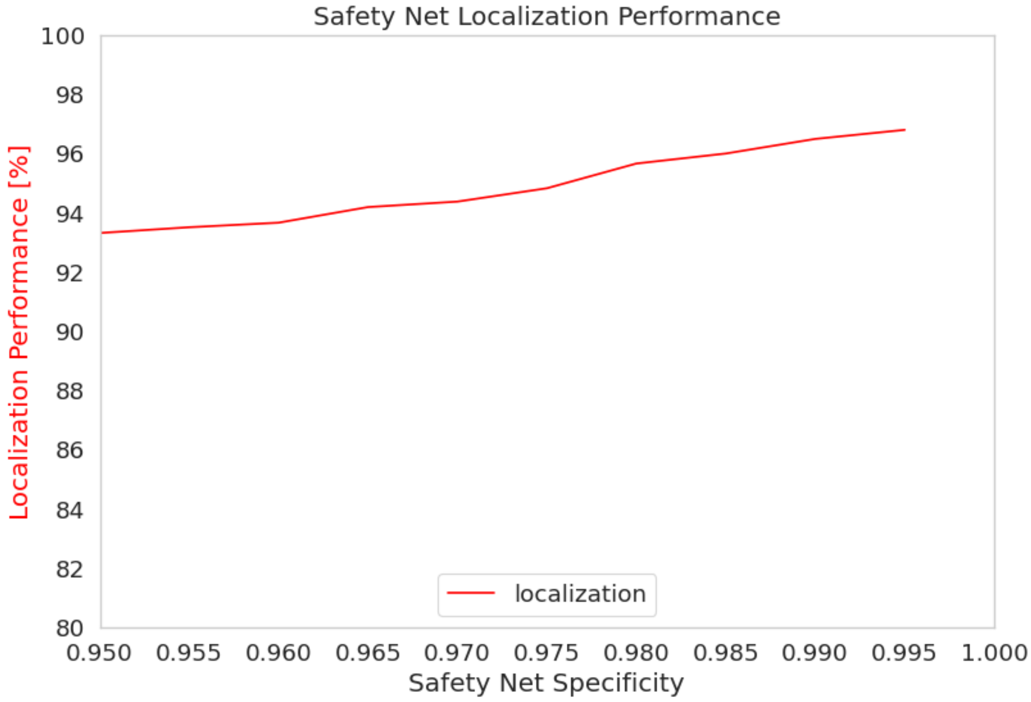

References

1. Kääb-Sanyal VH, Elisabeth. Jahresbericht Evaluation 2018: Deutsches Mammographie-Screening-Programm. Berlin, 2020.
2. Ovadia Y, Fertig E, Ren J, et al. Can you trust your model's uncertainty? Evaluating predictive uncertainty under dataset shift. arXiv preprint arXiv:190602530 2019.
3. Mansournia MA, Altman DG. Inverse probability weighting. BMJ (Clinical research ed) 2016; 352: i189.
4. Pinsky PF, Gallas B. Enriched designs for assessing discriminatory performance—analysis of bias and variance. Statistics in medicine. 2012 Mar 15;31(6):501-15.
5. Lundberg S, Lee S-I. A unified approach to interpreting model predictions. arXiv preprint arXiv:170507874 2017.
